# Precise in-field molecular diagnostics of crop diseases by smartphone-based mutation-resolved pathogenic RNA analysis

**DOI:** 10.1038/s41467-023-39952-x

**Published:** 2023-07-19

**Authors:** Ting Zhang, Qingdong Zeng, Fan Ji, Honghong Wu, Rodrigo Ledesma-Amaro, Qingshan Wei, Hao Yang, Xuhan Xia, Yao Ren, Keqing Mu, Qiang He, Zhensheng Kang, Ruijie Deng

**Affiliations:** 1 College of Biomass Science and Engineering, Healthy Food Evaluation Research Center, Sichuan University, Chengdu, 610065 China; 2 State Key Laboratory of Crop Stress Biology for Arid Areas, Northwest A&F University, Yangling, 712100 China; 3 MOA Key Laboratory of Crop Ecophysiology and Farming System in the Middle Reaches of the Yangtze River, College of Plant Science & Technology, Huazhong Agricultural University, Wuhan, 430070 China; 4 Department of Bioengineering, Imperial College Centre for Synthetic Biology, Imperial College London, London, SW7 2AZ UK; 5 Department of Chemical and Biomolecular Engineering, Emerging Plant Disease and Global Food Security Cluster, North Carolina State University, Raleigh, NC 27696 USA

**Keywords:** Bioanalytical chemistry, Biosensors, Microbiology techniques, Microbe

## Abstract

Molecular diagnostics for crop diseases can guide the precise application of pesticides, thereby reducing pesticide usage while improving crop yield, but tools are lacking. Here, we report an in-field molecular diagnostic tool that uses a cheap colorimetric paper and a smartphone, allowing multiplexed, low-cost, rapid detection of crop pathogens. Rapid nucleic acid amplification-free detection of pathogenic RNA is achieved by combining toehold-mediated strand displacement with a metal ion-mediated urease catalysis reaction. We demonstrate multiplexed detection of six wheat pathogenic fungi and an early detection of wheat stripe rust. When coupled with a microneedle for rapid nucleic acid extraction and a smartphone app for results analysis, the sample-to-result test can be completed in ~10 min in the field. Importantly, by detecting fungal RNA and mutations, the approach allows to distinguish viable and dead pathogens and to sensitively identify mutation-carrying fungicide-resistant isolates, providing fundamental information for precision crop disease management.

## Abstract (web summary)

On-site crop disease diagnostics is critical for precise application of pesticides. Here, the authors report an in-field molecular diagnostic tool for wheat pathogens using a nucleic acid amplification-free, gene mutation-resolved and smartphone-integrated genetic assay.

## Introduction

A sustainable food supply is burdened by the increasing global population^1^. Crop diseases caused by phytopathogens, mainly fungal pathogens, devastate crops and put food supplies at risk^2–4^. To combat crop pathogens, pesticides are overused, which has damaged the environment and soil health. However, despite two million tonnes of pesticides, the Food and Agriculture Organization reports an annual yield reduction of wheat and rice by 21.5% and 30.0%, respectively^5^. Crop fungal disease management is therefore crucial for food security, but remains challenging. Molecular diagnostic tools that can be routinely deployed in the field to provide the information about clonal lineage, virulence and drug-resistance of pathogens, should enable us to detect an infection at the early stage, precisely estimate the disease risk, and decide which fungicide to use, and when^6–8^. These tools are essential for crop disease control, but are currently unavailable.

Conventional phenotypic methods such as symptom observation, foliage machine vision and imaging approaches—including hyperspectral, thermographic, and infrared thermometric imaging technologies—have been successively employed to achieve non-invasive and in-field measurements of crop infection^9–14^. In most cases, however, these methods cannot identify the pathogen. Molecular diagnostics based on genotypic or immunological methods can offer information about pathogeny^15,16^. Nucleic acid-based methods, in principle, can cover the detection of any species of pathogens^17–20^. Besides, genetic information can illuminate the virulence and drug resistance features of phytopathogens^21,22^, which is vital for guiding the adoption of the optimal treatment strategy for particular diseases. An available low-cost in-field genotypic method could fill the gaps between molecular diagnostics and conventional crop disease monitoring. However, nucleic acid-based methods are currently complex, primarily due to the requirement for a nucleic acid amplification process. Nucleic acid assays developed to date are mostly restricted to labs, and are not applicable in the field.

Here, we demonstrate an in-field, rapid and cost-effective screening strategy for wheat pathogens with information about viability and drug-resistance using a nucleic acid amplification-free, gene mutation-resolved and smartphone-integrated genetic assay. The smartphone-based diagnostic tool has great potential advancing precision crop disease management.

## Results

### Assay principle

**Fig. 1:**
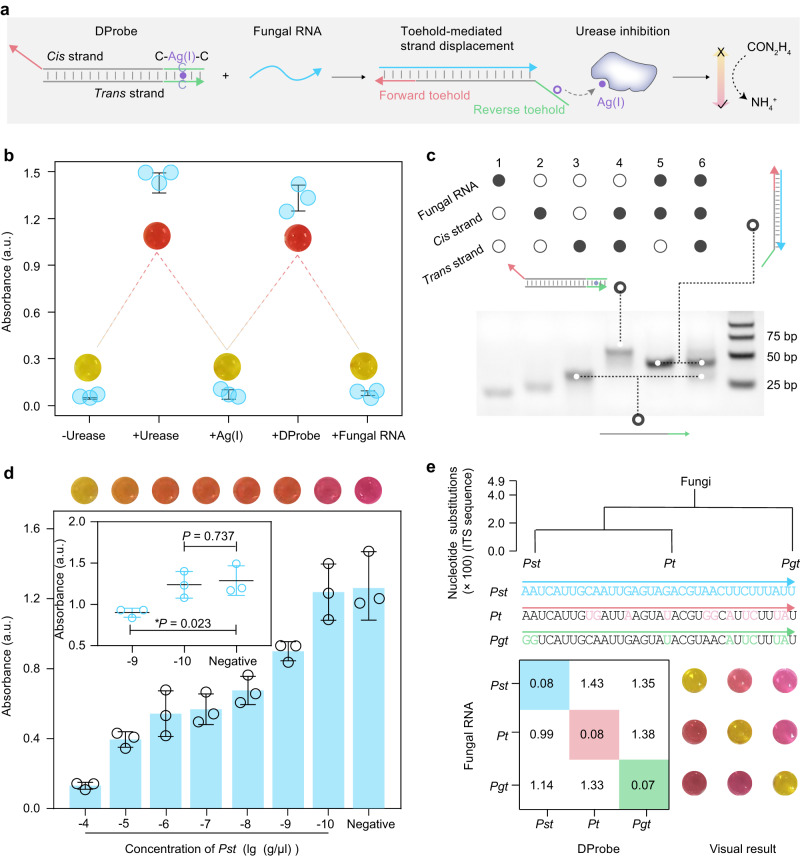
Visual detection of fungal RNAs based on the TMSD reaction and metal ion-mediated urease catalysis. **a** Schematic illustration of the colorimetric assay. The DProbe is a double-stranded DNA consisting of a *cis* strand and a *trans* strand that anneal to form a C-Ag(I)-C artificial base pair. Fungal RNAs can bind with the *cis* strand to release the *trans* strand and Ag(I) ion via the TMSD reaction. The driving force of the TMSD reaction derives from the hybridization between the forward toehold (pink domain) overhang and fungal RNAs, and the resistance force comes from the break of the reverse toehold (green domain) hybridization. The released free Ag(I) ion induced by fungal RNAs inhibits urease activity, reducing the production of NH_4_^+^, which can be visualized using of pH indicator phenol red. Based on this principle, the presence of fungal RNAs can be detected by the a colour change of the pH indicator. **b** Absorbance at 560 nm at each reaction of the assay. Inset: visual results of each reaction. Concentrations of DProbe, urease, urea, and phenol red were 100 nM, 1 nM, 500 mM and 250 μM. **c** Electrophoretic analysis of the TMSD reaction. Concentrations of DProbe, and fungal RNAs were 400 nM and 600 nM, respectively. To distinguish the DProbe from the hybrid of fungal RNAs and the *cis* strand in the electrophoresis ladder, 30 nt poly-T was added to the 5′ end of the *trans* strand. **d** Sensitivity estimation of the assay for detecting *Pst*. Visual results (upper) and absorbance (below) of each sample. lg (g/μl) indicates the negative logarithm of the concentration of *Pst*. **e** Discrimination of *Pst*, *Pt* and *Pgt*. The ITS sequences of *Pst*, *Pt* and *Pgt* (upper), absorbance at 560 nm (below left) and visual result (below right) for detecting different fungi. “a. u.” in (**b** and **d**) indicates arbitrary unit. Data in (**b** and **d**) represent means ± SD (*n* = 3). *P* values from Welch’s two-sided unpaired *t* test in (**d**): *P < 0.05. Source data are provided as a Source Data file.

The fungal RNA sequence is recognized based on a simple nucleic acid reaction, termed toehold-mediated strand displacement (TMSD), requiring a double-stranded DNA probe (the DProbe) (Fig. 1a and Supplementary Fig. 1a). The DProbe is a hybrid of the *cis* strand and the *trans* strand, and is designed with terminal domains called the forward and reverse toehold (shown in pink and green, respectively). The forward toehold is the domain in the *cis* strand that hybridizes with the fungal RNA and not with the *trans* strand. Conversely, the reverse toehold is the domain that hybridizes with the *trans* strand and not with the fungal RNA. There is one cytosine-Ag(I)-cytosine artificial base pair in the reverse toehold (Supplementary Fig. 1b). In the presence of fungal RNA, the TMSD reaction is initiated and facilitated via hybridization between the fungal RNA and the forward toehold, but is hindered by the disruption of the reverse toehold hybridization. Thus, the reaction can be precisely controlled via tuning the toehold domain. Fungal RNA fuels the TMSD reaction to release the *trans* strand and the Ag(I) ion from the DProbe. The presence of fungal RNAs is then indicated by urease-based ammonia production and a pH indicator, phenol red (which turns from yellow to red over the pH range 6.8 to 8.2). Urease catalyses the hydrolysis of urea to yield carbamate and ammonia (Supplementary Fig. 2); the production of ammonia contributes to a notable pH increase (to pH > 8.2), where upon phenol red acquires its red colour. Simultaneously, urease is highly sensitive to trace quantities of Ag (I) ion^23^; Ag(I) ion has a strong inhibitory effect on urease, causing phenol red to become yellow (pH <6.8). The presence of the fungal RNA releases Ag(I) ions to block urease, and can thus be visualized by the colour change of phenol red.

*Puccinia striiformis* (*Pst*) can cause a destructive disease of wheat, stripe rust, and significantly reduce wheat production worldwide^24,25^. We synthesized four short non-overlapping RNA sequences (22 nucleotides, nt) on the internal transcribed spacer (ITS) of *Pst*, to optimize the binding site in the ITS based on colour change of the assay (Supplementary Fig. 3 and Supplementary Table 1). The DProbe targeting the optimized binding site 3 was then used to test the assay principle. Urease catalysis and Ag(I) ion-induced urease inhibition were verified by measuring the absorbance of phenol red (Fig. 1b). The TMSD reaction was demonstrated using electrophoretic and fluorescence analysis (Fig. 1c, Supplementary Fig. 4 and Supplementary Table 2). For detecting long fungal RNAs from *Pst*, we found that lengthening the forward toehold from 8 nt to 15 nt facilitated the TMSD reaction, and improved assay’s response to *Pst* (Supplementary Fig. 5).

We then evaluated the assay performance for detecting *Pst*. A dilution test of *Pst* showed that the assay can detect 1.0 ng/μL *Pst* without a nucleic acid amplification procedure (Fig. 1d) (Welch’s *t* test: *P <  0.05). Neither extraction of nucleic acid from *Pst* infected wheat leaves nor mixing *Pst* RNAs with wheat leaf RNAs significantly affected the absorbance of the assay compared to only *Pst* RNAs (Supplementary Fig. 6), and the presence of wheat RNA matrix did not compromise the sensitivity for detecting *Pst* (Supplementary Fig. 7).

Congeneric pathogenic fungi can cause similar infection symptoms and have been overlapping geographical distribution. We tested whether the assay could discriminate among three congeneric rust fungi, *Pst, Puccinia graminis* (*Pgt*) and *Puccinia triticina* (*Pt*). The ITS sequences of *Pgt* and *Pt* are similar to that of *Pst* (Fig. 1e). Three DProbes were designed to target the ITSs of *Pst*, *Pgt* and *Pt*. We mixed each set of RNAs extracted from these pathogenic fungi, and each could be detected using its corresponding DProbe without cross-interference. Besides rust fungi, *Blumeria graminis* (*Bgt*), *Fusarium graminearum* (*Fg*) and *Rhizoctonia cerealis* (*Rc*) responsible for yield reduction of wheat^26^, they cause the diseases powdery mildew, fusarium head blight and wheat sharp eyespot, respectively. By aiming for the ITS sequence of the six fungi (Supplementary Table 3), the assay allowed visual detection of each species (Supplementary Fig. 8).

### Multiplex detection of fungal pathogens using colorimetric papers

**Fig. 2:**
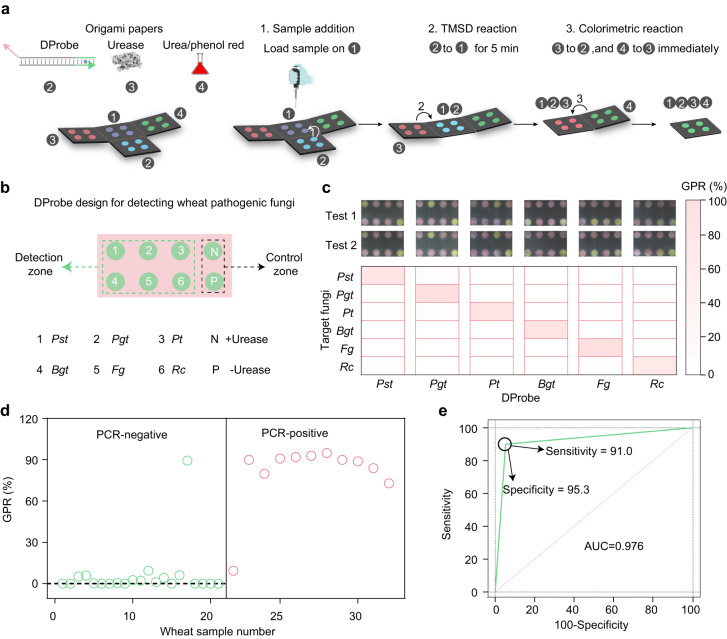
Multiplexed visual detection of fungal pathogens. **a** Integration of each reaction of the assay using a paper-folding strategy. **b** Paper designs for detecting six wheat pathogenic fungi. **c** Multiplexed detection of six fungi using the colorimetric paper. **d** GPR values obtained by testing the 32 field-collected wheat leaf samples. **e** Comparison of results for detecting *Pst* infection in 32 wheat samples in (**d**) using the colorimetric assay and qPCR. Concentrations of DProbe, urease, urea, and phenol red were 100 nM, 1 nM, 500 mM and 250 μM. Source data are provided as a Source Data file.

To facilitate in-field detection, we integrated each assay reaction using a paper folding strategy termed origami. Wax printing was utilized to prepare the origami paper with defined detection spots via the formation of hydrophobic barrier^27^. The procedures are shown in Supplementary Fig. 9. Reagents including DProbe, enzyme, and pH indicator were then loaded on each page of the paper (Fig. 2a). The folding procedure sequentially initiated the TMSD reaction and colour reporting reaction, and yielded a folded paper to be imaged by the camera on a smartphone. We developed an algorithm to locate the detection spot and defined a green pixel ratio (GPR) value to record the colour response of phenol red towards fungal RNAs. GPR value was defined as the portion of pixels with positive grey value in the green channel within the detection spot. The image processing method is illustrated in Supplementary Fig. 10. We found that the origami paper with a large pore size or surfactant modification yielded improved sensitivity, colour consistency and uniformity of the colorimetric response via the paper-folding strategy (Supplementary Fig. 11, Supplementary Fig. 12, Supplementary Note 1). In addition, the robustness of the detection of *Pst* using the origami papers related to batches of origami papers and operators has been tested (Supplementary Fig. 13). Colour calibration has been demonstrated to be able to improve the robustness of the detection using different smartphones (Supplementary Fig. 14).

By designing the origami paper with eight sample-loading sites, six pathogenic fungi (*Pst, Pgt, Pt, Bgt, Fg* and *Rc*) can be in parallel detected (Fig. 2b). The result showed that all six fungi could be distinguished without cross-interference using one origami paper loaded with their congenetic probes (Fig. 2c). The colorimetric assay using the origami papers yielded a comparable sensitivity compared to that proceeded in solution, and allowed to detect as low as 1 ng/μL *Pst* (Supplementary Fig. 15). We further used the assay to detect fungal pathogens in infected wheat samples. The infection of *Fusarium culmorum* that causes fusarium crown rot can be indicated via the colour change of the origami papers (Supplementary Fig. 16). To further evaluate the accuracy of the assay, thirty-two wheat leaf samples collected from different fields in China were tested (Supplementary Figs. 17, 18). The samples were analysed in parallel using quantitative PCR (qPCR) and the colorimetric paper-based assay. The assay showed an agreement of 95.3% positive prediction, and 91.0% negative prediction when compared to qPCR in the test of the infection of *Pst* (Fig. 2d, e, Supplementary Table 4, 5). Besides pathogenic fungi, we explored the assay to detect a plant pathogenic bacterium, *Pseudomonas syringae* (Supplementary Fig. 19) and a crop pathogenic virus, barley stripe mosaic virus (Supplementary Fig. 20), and demonstrated that the assay is capable to diagnose the infection of not only fungi but also viruses and bacteria, showing the potential for the broad applicability for in-field detection of crop diseases.

### Early diagnosis of fungal infection

**Fig. 3:**
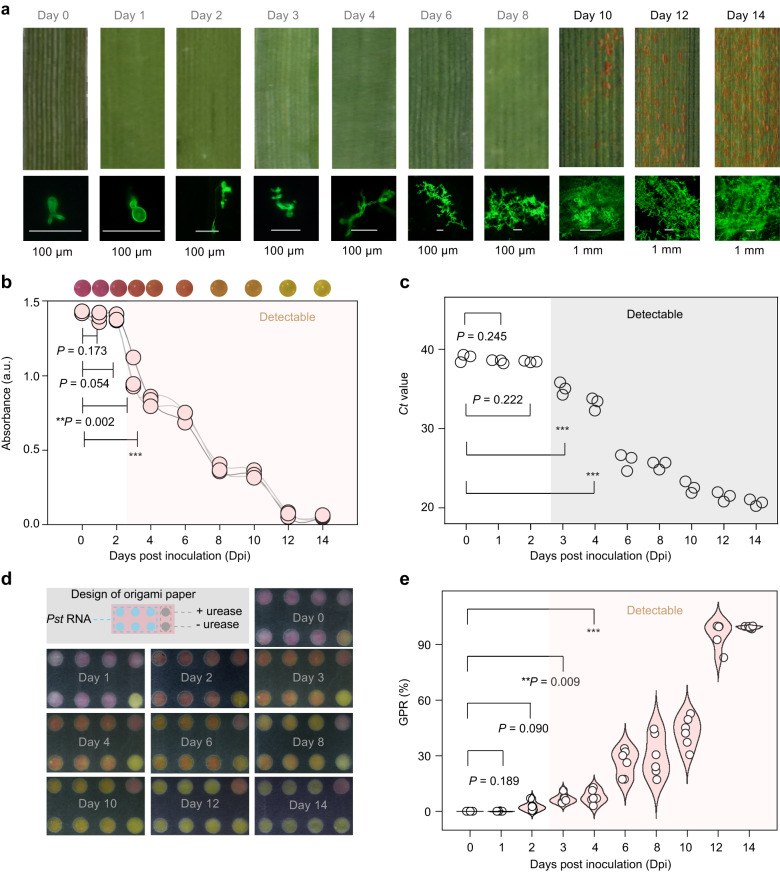
Early diagnosis of fungal infection. **a** Phenotyping of wheat leaves during 14 days after infection with *Pst*. Wheat leaves were detached and photographed using a smartphone (upper), and fluorescence micrographs of the fungal structures were obtained (below). Fungal structures were stained with wheat germ agglutinin. **b** Visual result (upper) and absorbance at 560 nm (below) of the test of wheat leaves infected for 0–14 days. **c** Detection of *Pst* by qPCR of wheat leaves infected for 0–14 days. **d** Paper design for parallel detection of *Pst* in infected wheat samples. **e** Detection using colorimetric paper of *Pst* from wheat leaves infected for 1–14 days. Concentrations of DProbe, urease, urea and phenol red were 100 nM, 1 nM, 500 mM and 250 μM. Shadings in (**b**–**d**) indicated the positive detection of *Pst* infection. Data in (**b**) and (**c**) are means ± SD (*n* = 3). Data in (**e**) are means (*n* = 6). *P* values from Welch’s two-sided unpaired *t* test in (**b,** **c** and **e**): *P < 0.05, **P < 0.01, ***P < 0.001. Source data are provided as a Source Data file.

Early detection of crop infection can dramatically alleviate crop yield reduction and reduce fungicide use. We tested the assay for early diagnosis of *Pst* infection. Figure 3a shows the observed phenotype of wheat leaves for during two weeks after infection. Spores are visible by the naked eye on day 10, demonstrating that wheat infection can be recognised after 10-day-infection by symptom observation. *Pst* in infected wheat leaves was stained using wheat germ agglutinin^28^. Histological observation also showed the rapid spread of *Pst* in the leaves by10 days after inoculation.

Nucleic acid was extracted from the infected leaves, and analysed by the colorimetric assay and qPCR. RNA extracted from the wheat samples on day 3 triggered a sharp drop of the absorbance signal in the colorimetric assay (Fig. 3b) (Welch’s *t* test: *P <  0.05). *Pst* infection can thus be identified after 3-day infection using the colorimetric assay. The qPCR result yielded positive detection of *Pst* from the 3-day-infected wheat samples (Welch’s *t* test: ***P <  0.001) (Fig. 3c, Supplementary Fig. 21). It was estimated that 64.29 ng/μL *Pst* was present in the 3-day-infected wheat samples (Supplementary Fig. 22). The paper-based strategy was further applied for parallel detection of *Pst*-infected wheat samples (*n* = 6). *Pst* infection was ascertained after 3 days using the colorimetric paper (Fig. 3d) (Welch’s *t* test: **P <  0.01). The paper-based assay using optimized origami paper yielded an increased GPR value for testing *Pst* from the wheat sample infected for 3 days and a reduced variation in duplicate detection (Supplementary Fig. 23), facilitating the robustness of positive detection of early infection by *Pst*. In addition, we inoculated wheat leaves with *Pst* in a dilution series. The colorimetric assay allowed to detect low-level *Pst* that did not cause observable infection symptom in the wheat leaves infected for 14 days (Supplementary Fig. 24). Collectively, the colorimetric assay showed a capacity for early detection of *Pst* infection comparable to qPCR, and advanced the identification of an infection by 7 days compared to symptom observation.

### Viable fungus detection facilitates prediction of disease occurrence and severity

**Fig. 4:**
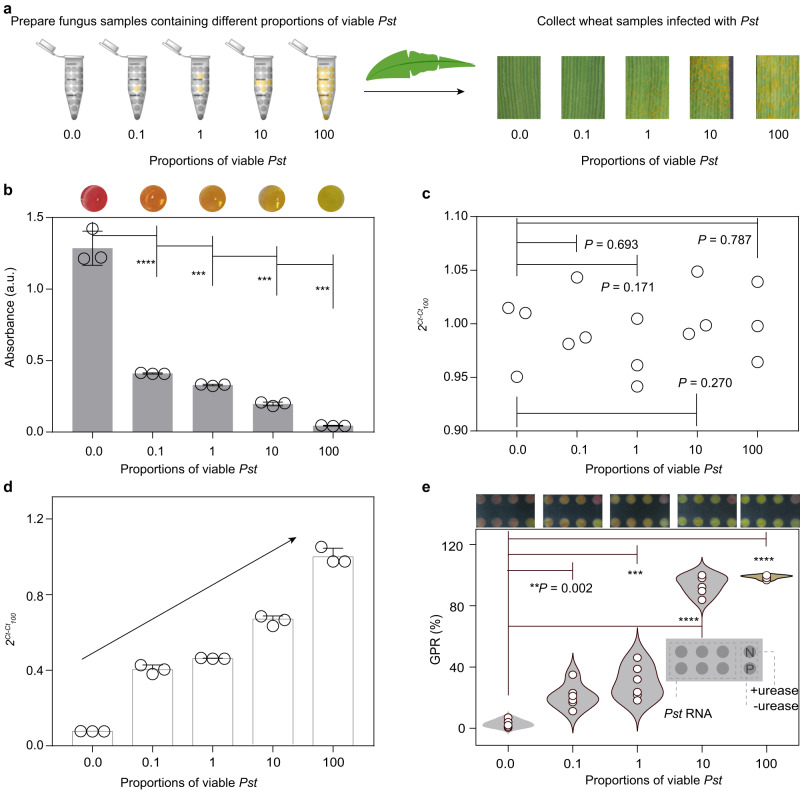
Detection of viable fungi increases precision for disease risk prediction. **a** Scheme for the inoculation of wheat with an equal quantity of *Pst* mixtures containing 0%, 0.1%, 1%, 10% and 100% viable fungi, and symptom observation of infected wheat leaves after 14-day infection. **b**, **c**, Detection of *Pst* in samples in (**a**) using the colorimetric assay (**b**) and qPCR (**c**). Measurement of *Pst* in 14-day-infected wheat leaves using qPCR (**d**) and the colorimetric assay (**e**). Paper design; upper in (**e**), photographs of origami papers. *Ct*_*100*_ means the *Ct* value of *Pst* mixtures containing 100% viable fungi (in **c**) or the *Ct* value of wheat samples infected with *Pst* mixtures containing 100% viable fungi (**e**). Data in (**b**–**d**) are means ± SD (*n* = 3). Data in (**e**) are means (*n* = 6). *P* values from Welch’s two-sided unpaired *t* test in (**b,** **c** and **e**): **P < 0.01, ***P < 0.001, ****P < 0.0001. Source data are provided as a Source Data file.

Disease cycles comprise four stages: dormancy, reproduction, dispersal and pathogenesis^29^. Pathogens may be present on residues left in the field, in soil, and on weeds and tools. Importantly, only viable pathogenic fungi in dormancy will be activated in favourable conditions and enter the reproduction stage. Therefore, it is essential to devise strategies to distinguish viable fungi in total fungal samples to prevent disease circulation. Current DNA-targeting methods, such as qPCR, usually fail to distinguish viable pathogens from dead ones for the long-term persistence of DNA in dead microbial cells. RNA, however, is rapidly degraded in dead cells, and so, methods that target RNAs, such as the colorimetric assay, should specifically detect viable pathogens^30,31^. We therefore tested the feasibility of viable fungus detection using *Pst* mixtures containing viable and dead spores, in which the portion of viable spores was 0%, 0.1%, 1%, 10% and 100% (Fig. 4a). Nucleic acid was extracted from equal quantities of these *Pst* mixtures, and analysed using the colorimetric assay and qPCR. The results showed that absorbance signals attenuated gradually with the increase of viable fungi in both the absence and presence of wheat RNA matrix (Fig. 4b, Supplementary Fig. 25), indicating that the colorimetric assay can reliably indicate the amount of viable fungus. In contrast, there was no significant difference among *Pst* mixtures using qPCR (Fig. 4c, Supplementary Fig. 26).

*Pst* mixtures with different proportions of viable spores were utilized to inoculate wheat. Due to the low abundance of *Pst*, the *Ct* values for qPCR measurement of *Pst* infection at 0 day were all close to 40 (Supplementary Fig. 27). Fourteen days after the infection, leaves inoculated with *Pst* mixtures containing 0% and 0.1% viable fungi showed no obvious symptoms, but when the content of viable fungi increased to 1%, the leaves turned yellow and were loaded with observable spore piles (Fig. 4a). Measurements using qPCR and the colorimetric assay indicated that the amount of both total and viable *Pst* fungus in infected leaves increased in accordance with the proportion of viable fungus in the *Pst* mixture used for inoculation (Fig. 4d, e and Supplementary Fig. 28). This result indicated that the occurrence and severity of stripe rust were related specifically to the quantity of viable fungus, rather than the total quantity of all fungus. Detection of viable fungal pathogens is highly important, given that a large proportion of fungal cells may die through winter and summer. These fungi cannot cause effective crop infection. Because it can detect viable fungi, the colorimetric assay allows for a more precise prediction of disease occurrence and severity compared to methods that cannot distinguish viable pathogens from dead ones.

We also investigated the defence response of wheat towards dead *Pst*. Dead spores slightly increased the expression of two pathogenesis-related genes, PR1 and PR2 (Supplementary Fig. 29)^32,33^, but pre-inoculation with dead spores did not significantly change *Pst* biomass after 12, 24 or 48 h of infection with viable spores (Supplementary Fig. 30, Supplementary Note 2). The result indicates that dead pathogenic fungi do not elicit a defence response that can efficiently inhibit pathogen infection.

### Identification of fungicide-resistant isolates

Fungicides are intensively used to prevent and treat crop diseases. The emergence of fungicide-resistance has become a severe issue. Identifying fungicide-resistant isolates can instruct us to choose an effective fungicide or other treatment strategies^34^. Mutations leading to conformational changes in the drug target site are the main cause of fungicide-resistance in pathogenic fungi. CYP51, one of the cytochrome P450 monooxygenases, acts on fungal invasive growth, hypha formation and virulence. Inhibitors that target CYP51 serve as key antifungal agents^35,36^. The point mutation Y134F in CYP51 was found to be associated with a significant degree of triadimefon resistance in *Pst* isolates^37^.

**Fig. 5:**
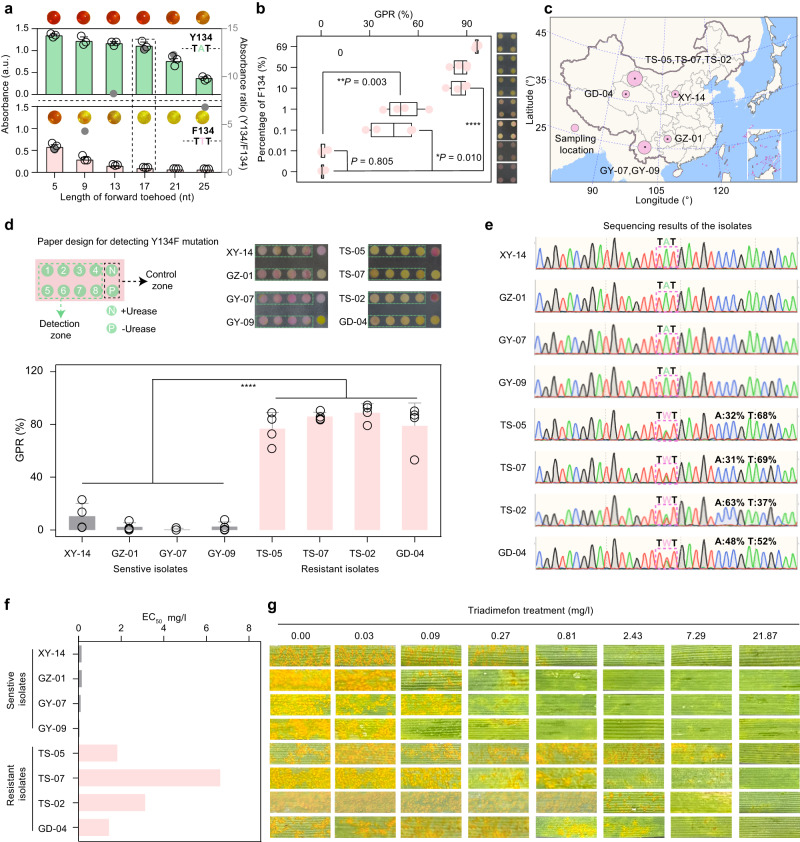
Identification of mutation-induced fungicide-resistant isolates. **a** Optimization of the DProbe for detecting the Y134F mutation. The highest discrimination of F134 to Y134 was obtained using a DProbe with 17-nt forward toehold (dotted box) **b** Detection of Y134F-mutated *Pst* in a dilution series. **c** Collection sits of eight *Pst*-infected wheat samples in China. **d** Detection of the Y134F mutation in the collected *Pst* isolates. Paper design (upper left), photographs of origami papers (upper right) and GPR values (below) testing samples in **c e**, Sequencing results of the *Pst* isolates. **f** EC_50_ values of the eight *Pst* isolates towards triadimefon. **g** Phenotyping of wheat treated with triadimefon. Data in (**a**) and (**d**) are means ± SD (**a**, *n* = 3; **b**, **d**, *n* = 4). The bottom and top lines of the box in (**b**) represent the first and third quartiles respectively, the middle line in the box indicate the median value. The whisker lines indicate the minimum and maximum value within 6 independent testes, respectively. Map in (**c**) is downloaded from http://bzdt.ch.mnr.gov.cn/index.html with the Map Audit Number: GS (2019) 1836. *P* values from Welch’s two-sided unpaired *t* test in (**b**) and (**d**): *P < 0.05; **P < 0.01; ****P < 0.0001. Source data are provided as a Source Data file.

Via competitive hybridization to hinder binding with non-target RNAs, an assay based on TMSD showed promise for detecting single-nucleotide mutations (SNMs) in RNAs. The principle of TMSD for identifying SNMs is illustrated in Supplementary Note 3. To maximize discrimination between the mutated RNA (F134 RNA) and the non-target wild type RNA (Y134 RNA), with a single-nucleotide difference, the TMSD reaction should be optimized by tuning the toehold length of the DProbe, which allows to block non-specific hybridization induced by the wild RNA while the DProbe/mutated RNA hybrid still forms. We designed DProbes targeting the Y134F mutation with a fixed 7-nt reverse toehold and forward toeholds ranging from 5 nt to 25 nt (Supplementary Fig. 31a, b). Fluorescence analysis of the TMSD reaction using a fluorophore and quencher-modified DProbe showed that DProbes with forward toehold lengths of 5, 9, 13, and 17 -nt can effectively distinguish F134 RNA from Y134 RNA. Long forward toeholds (21 nt and 25 nt) yielded a low discrimination capacity for the mutation because both F134 RNA and Y134 RNA can efficiently fuel the TMSD reaction. In contrast, shorter toehold length led to a lower displacement efficiency for both F134 RNA and Y134 RNA (Supplementary Fig. 31e). Electrophoretic analysis confirmed these results (Supplementary Fig. 31f). Based on the colorimetric reaction of the assay (Fig. 5a), the highest ratio of the absorbance of F134 RNA to that of Y134 RNA was achieved using the DProbe with a 17-nt forward toehold. Using this optimized DProbe, a dilution experiment with the mutated isolate indicated that, in either the absence or the presence of wheat RNA matrix, the assay could detect as little as 0.1% mutated isolate in a background of 99.9% wild type (Fig. 5b, Supplementary Fig. 32). The assay can thus detect low-abundance single-nucleotide mutations and should therefore be useful for finding rare fungicide-resistant isolates.

We collected eight *Pst*-infected wheat samples from field sites in China (Fig. 5c). Using the above assay, we found that four isolates (XY-14, GZ-01, GY-07 and GY-09) from these samples were Y134F mutation-free, and four isolates (TS-05, TS-07, TS-02 and GD-04) had the Y134F mutation (Fig. 5d), which indicated the possibility of triadimefon resistance in the latter four isolates. *Pst* isolates from the eight wheat leaves were collected and sequenced. XY-14, GZ-01, GY-07 and GY-09 were homozygous wild type; and TS-05, TS-07, TS-02 and GD-04 were heterozygous Y134F mutants (Fig. 5e). Responses of eight *Pst* isolates to triadimefon showed that TS-05, TS-07, TS-02 and GD-04 had a much higher EC_50_ towards triadimefon than other isolates (Fig. 5f, g, Supplementary Fig. 33, and Supplementary Table 6). These results demonstrate the feasibility of the colorimetric assay for identifying mutation-carrying fungicide-resistant isolates.

### Smartphone-assisted in-field diagnosis

**Fig. 6:**
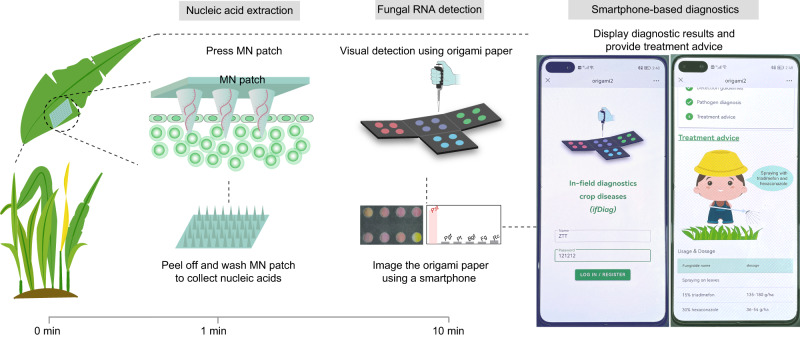
Smartphone-based in-field diagnosis. Nucleic acid extraction from wheat leaves is achieved using an MN patch, and fungal RNAs are detected using origami papers. Diagnostic results are displayed by a smartphone app. Based on the diagnostic results, the app can offer treatment strategies for the crop disease.

To explore the ‘sample-to-answer’ in-field use of the assay, we integrated the assay with rapid nucleic acid extraction strategies, smartphone attachment and a mobile application program (app) (Fig. 6, Supplementary Fig. 34).

Nucleic acid extraction is a bottleneck for on-site or in-field nucleic acid tests, due to the complex setups involved that include centrifugation and chemical reagents. First, we explored a simple strategy using quartz sand to grind wheat leaf samples and release nucleic acid (Supplementary Fig. 35), and found that the presence of *Pst* can be detected due to the colour change compared to samples without *Pst*. However, pigments in leaves can induce colour deviation in the papers, and consequently the quantification of GPR values was not stable. As an alternative, we then synthesized a microneedle (MN) patch for nucleic acid extraction (Supplementary Figs. 36, 37). The MN patch, made of polyvinyl alcohol, has been used to extract DNA from leaf tissues^38^. The patch can penetrate the wheat leaf and break cell walls to isolate nucleic acids. Via a rapid swelling through absorption of water molecules, the patch absorbs and concentrates nucleic acids on the needle tips. MN patch extraction yielded high-purity nucleic acid, but extracted less nucleic acid, due to the relatively low sample volume, than the Trizol-based method (Supplementary Fig. 38). Nevertheless, the use of an MN patch accomplishes nucleic acid extraction within 1 min via a single press action; no instruments or chemical reagents are needed, thus facilitating nucleic acid tests for in-field diagnostics for crop diseases.

By designing a smartphone attachment, the colorimetric detection of fungal RNAs could be completed by three simple pull operations, and the risk of contamination was avoided (Supplementary Fig. 39). Furthermore, an app was designed for the graphical user guidance and the display of diagnostic results (Supplementary Fig. 40). Notably, the app can also provide treatment advice based on the diagnostic results (Supplementary Fig. 41, Supplementary Table 7), which is an essential feature for effective crop disease control.

## Discussion

We have reported an in-field molecular diagnostic platform for crop diseases based on a cheap (~$0.30 per test), rapid (~10 min), multiplexed (6 pathogens or more) and mutation-resolved genetic assay. Compared to currently available genotypic methods, such as qPCR, the proposed assay is nucleic acid amplification-free and colour-readable, which simplifies operation and result readouts while eliminating the need for dedicated instruments, thus allowing in-field use. It is expected to be used by farmers, enabling them to detect crop infection at a very early stage by screening potential pathogens, and to obtain instructions about treatment protocols from smartphones.

Crop disease diagnosis at the early stage can dramatically reduce fungicide use while alleviating crop yield reduction. Despite its lack of nucleic acid amplification, the assay is enough to achieve early detection of fungal infection. Using a metal ion-mediated urease catalysis reaction, the recognition of fungal RNAs is amplified to become a colour-readable signal, yielding a limit of detection of 1.0 ng/μL *Pst*. Due to this high sensitivity, the assay allowed to identify 3-day infection by *Pst*, advancing the identification by 7 days compared to symptom observation, by 3 days compared to hyperspectral imaging^39^, and to a period comparable to the sensitive genotypic method, qPCR, for early detection of *Pst* infection. Besides, the diagnostic accuracy for wheat stripe rust using the colorimetric assay was an improvement of about ~6% compared to the reported hyperspectral imaging technique^39^.

Identification of the specific crop pathogen is critical for choosing which fungicide to use, but is practically unachievable by phenotypic methods such as foliage machine vision, or by hyperspectral or thermographic imaging approaches. Screening pathogens requires a multiplexed assay that can distinguish the genotypic, immunological or chemical differences among pathogens and encompass the detection of potential pathogens. The colorimetric paper was patterned for multiplexed detection of fungal RNAs, and we demonstrated the parallel detection of six prominent fungi that can infect wheat (*Pst, Pgt, Pt, Bgt, Fg* and *Rc*). The multiplexing capacity of the assay can be expanded by printing more reagent loading sites. In particular, given that the DProbe can be reprogrammed to recognise specific fungal RNAs, the assay can, in principle, diagnose any crop pathogen of interest, thus offers a programmable multiplexing capacity to cover all potential pathogens that infect the cultivated crop.

Fungal pathogens can reside in soil and on weeds, but many of them cannot survive through summer and winter. Only viable ones will be activated in favourable conditions, to infect crops and cause plant diseases. Therefore, detection of viable pathogens is important for estimating infection and disease risk. By detecting fungal RNAs, the proposed assay allows to differentiate viable and dead fungi. In contrast, qPCR cannot distinguish dead from viable fungi. We showed that an increase in the proportion of viable *Pst* led to a more severe symptom of wheat stripe rust, indicating that the occurrence and severity of stripe rust correlates with the quantity of viable *Pst*, rather than total *Pst*. Although disease occurrence cannot be determined by pathogen presence alone, because the host plant and environment are also key factors^3^, the detection of viable pathogens that are active, rather than pathogens, that are dead, should increase the prediction accuracy of disease occurrence and severity. For example, the estimated abundance of viable fungi in soil obtained via the assay may be highly valuable for predicting the occurrence of soil-borne crop diseases.

Knowing the fungicide-resistance of fungal pathogens can further help us to choose the right fungicide or other intervention strategies. This is exemplified by the identification of antibiotic-resistant infectious diseases in humans, which contributes to reducing the mortality and morbidity rate of nosocomial infections^40^. Drug resistance is usually caused by genetic mutations. Only molecular methods are potentially applicable for discriminating the subtle genetic difference involved. However, PCR lacks the sequence specificity to identify resistance caused by point mutations, while sequencing techniques are currently impractical in the field, and are also costly. By using a strand displacement reaction to identify fungal RNAs, the proposed assay allows to discriminate single-nucleotide mutations. We demonstrated its identification of a single-nucleotide mutation, Y134F, that is associated with resistance to the fungicide triadimefon. Moreover, the assay could detect down to 0.1% mutated *Pst* in an otherwise wild-type population, indicating its capacity to find rare fungicide-resistant pathogens.

In particular, fungal pathogen screening, viable pathogen detection and drug-resistance identification are now achievable in-field by simply using a low-cost test paper and a smartphone, greatly advancing plant disease diagnostics. Nucleic acid extraction is simplified using an MN patch, allowing this step to be completed within 1 min. Simple pull operations allow the users to accomplish the colorimetric detection of fungal RNAs by means of a 3D-printed smartphone attachment, and the sample-to-result test for wheat diseases can be finished in 10 min in the field. The smartphone app can recommend intervention strategies based on the diagnostic results. Therefore, the end-users without any knowledge about phytopathology can be reliably guided to precisely treat the crop infection. Besides, the colorimetric assay only requires cheap reagents such as urease and non-chemically labelled DNA probes, yielding an inexpensive test covering six wheat fungal pathogens (estimated to be US $0.30). The low cost of tests and wide availability of smartphones should render the assay to be an abundantly available, regularly applicable in-field diagnostic tool for crop diseases. In comparison, a nanopore sequencing-based method, termed MARPLE has been utilized for high-throughput and point-of-care detection of strain-level fungal pathogens and fungicide resistance with single-nucleotide resolution, but involves the nucleic acid amplification process, the use of PCR instrument and MinION sequencer^41^, thus increasing the assaying time (to be 48 h), complexity and the costs for the tests.

We demonstrate a proof-of-concept for the in-field diagnosis of fungal pathogens and their fungicide resistance, the detection performance of the assay could be further improved. First, the colorimetric paper-based assay has been used for the qualitative and semi-quantitative measurement of different pathogens, yet it is, currently, not feasible for quantitative detection. Paper-based colorimetric readout yields colour nonuniformity due to the variation of reagent diffusion^42^, and in most cases, it has not been used for target quantification. We explored the optimization and modification of paper substrates to alleviate the data nonuniformity. Besides, the development of image algorithms for colour calibration^43^ or rescaling^44^ and the use of deep learning approach^45^ show the promise to fuel paper-based colorimetric assays to be a quantitative assay. Second, although the assay has been used for determining the resistance of *Pst* towards triadimefon via detecting genetic mutations, gene mutation markers that can indicate drug resistance are still lacking, particularly for crop pathogens^46^. Thus, the assay can currently identify only a very small proportion of fungicide-resistant fungi, but this coverage will be improved as new drug-resistance genetic markers are discovered. With the development of new fungicides, as well as the emergence of new resistant fungi, the tools to identify fungicide resistance information will become increasingly important for precision plant disease control. In addition, microneedle-based nucleic acid extraction is rapid and simple, but has been only tested with leaf samples, a tough microneedle will be needed to extract nucleic acids from the samples with relatively hard surfaces such as roots.

The work advances molecular diagnostics for crop diseases via achieving in-field nucleic acid tests with single-nucleotide resolution. The application of the in-field diagnostic tool covering the detection of pathogenic fungi, viruses and bacteria is of potential to facilitate efficient crop disease management, reduce the use of pesticides, and contribute to sustainable agriculture via alleviating crop diseases in precise plant diagnostics.

## Methods

### Oligonucleotides

DNA oligonucleotides (Supplementary Table 1–3, 5) were ordered from Sangon (Shanghai, China). 6-carboxyfluorescein and Black Hole Quencher 1 modified DNA oligonucleotides, RNA oligonucleotides were purchased from Takara (Beijing, China). RNA oligonucleotides and chemically modified DNA oligonucleotides were purified via HPLC. DNA oligonucleotides were purified by PAGE. DNA and RNA oligonucleotides were dissolved in molecular biology grade water (cat. no. 46-000-CM, Corning) to prepare stock solutions with a concentration of 100 μM.

### DProbe preparation

DProbes were synthesized by annealing 5 μL *cis* strand (3 μM), 5 μL *trans* strand (3 μM) and 5 μL AgNO_3_ (3 μM) in 15 μL NaNO_3_ (1 M) at 90 °C for 5 min, followed by incubation at 25 °C for 25 min, and kept at 4 °C prior to use. Labelled DProbes were used to verify the formation of the TMSD reaction, and the fluorescence was measured using a Synergy H1 microplate reader and analyzed using Gen5 CHS 3.08 system. The excitation wavelength was 480 nm, the emission wavelength was 520 nm.

### Pathogen isolates

*Puccinia striiformis* (*Pst*) (CYR32), *Puccinia triticina (Pt)* (XJ-7), *Puccinia graminis* (*Pgt*) (HQM), *Blumeria graminis* (*Bgt*) (E09) and *Fusarium culmorum* (*F. culmorum*) were provided by Dr. Jie Zhao, College of Plant Protection of Northwest A&F University (Yangling, China). *Rhizoctonia cerealis* (*Rc*) (R0301) was provided by Dr. Li Wei, Institute of Plant Protection, Jiangsu Academy of Agricultural Sciences (Nanjing, China). *Pseudomonas syringae* pv. *tomato* DC3000 (*P. syringae pv. tomato* DC3000) was kindly provided by Dr. Honghong Wu, Huazhong Agricultural University, (Wuhan, China). Barley stripe mosaic virus (BSMV) was provided by Dr. Qiang Xu, Sichuan Agricultural University (Chengdu, China). *Fusarium graminearum* (*Fg*) (CICC 2697) was purchased from the China Centre of Industrial Culture Collection.

### Wheat cultivation and fungal inoculation

For *Pst* inoculation, wheat cultivar Suwon 11 was first grown in the greenhouse under 16 °C with 16-h light and 10 °C with 8-h dark. Once the wheat had grown to the two-leaf stage, the second leaf was inoculated with *Pst* spores and the plant was placed at 10 °C under saturated humidity with 24-h dark. The plant was then returned to normal conditions (16 °C with 16-h light and 10 °C with 8-h dark).

A soil mixing method was used for *F. culmorum* inoculation^47^. The mycelium blocks were placed on a millet culture medium and incubated at 25 °C for five days. When the surface of the millet was covered with mycelium, the cultivation was stopped and dried for later use. Sterile soil and millet culture medium were mixed evenly in a ratio of 125:1 to produce fungal soil. The mixture was transferred in plastic flower pots containing 200 g of fungal soil, plant ten healthy wheat seeds, and located in a greenhouse. The greenhouse temperature should be controlled at 25 ± 2 °C during the day and 20 ± 2 °C at night under natural light. Wheat samples were collected after 30-day infection for analysis.

### Barley cultivation and BMSV inoculation

BMSV-negative wheat was grown in an environmental chamber at 16–14 °C with a 16 h/light: 8 h/dark photoperiods. When the plants had grown to the two to three-leaf stage, BSMV were inoculated onto the second leaves from the bottom of wheat^48^. The seedlings were moisturized at saturated humidity for 24 h without light and then placed in an incubator at 25 °C for nine days before the phenotype was manifestation^49^.

### Arabidopsis cultivation and *P. syringae pv. tomato* DC3000 inoculation

Arabidopsis seeds were sown in soil mix and grown in growth chamber with the following settings: 22 °C for 10-h light and 20 °C for 14-h dark, 150 μmol m^−2^ s^−1^ light intensity, 70% relative humidity. Then, 4-weeks old *Arabidopsis* plants were infiltrated with solution containing *P. syringae* pv. tomato DC3000 (OD_600_ 0.001). After 3-h inoculation under room light condition, plants were transferred back to the growth chamber. Arabidopsis leaves were collected for test after another 3-day infection.

### Nucleic acid extraction

Fungi were first placed in 2-mL microcentrifuge tubes, frozen in liquid nitrogen, and powdered using a SCIENTZ-48 tissue grinder (Ningbo Scientz Biotechnology). DNA was extracted using an Ezup Column Fungi Genomic DNA Purification Kit (cat. no. B518259-0050, Sangon Biotech). Briefly, 200 μL Buffer Digestion, 2 μL β-Mercaptoethanol and 20 μL Proteinase K were added and incubated at 56 °C for 1 h to lyse the cells. Subsequently, 100 μL Buffer PF was added and stored at −20 °C for 5 min, followed by centrifuging at 12,000 × *g* for 5 min at 4 °C. The supernatant was transferred to a new adsorption column, mixed with 200 μL Buffer BD, 200 μL ethyl alcohol, and centrifuged at 9500 × g for 1 min. Then, 500 μL PW solution and 500 μL Wash solution were added in turn, followed by centrifuging at 12,000 × g for 2 min at 4 °C. Finally, DNA was resuspended in 50 μL H_2_O and used for qPCR.

RNA was extracted using TRIzol Reagent (cat. no.15596018, Thermo Fisher Scientific). 1 mL of which was added into the ground fungi and incubated at room temperature for 5 min. Then, 200 μL chloroform was added and vibrated for 15 s, followed by centrifuging at 12,000 × *g* for 15 min at 4 °C. The aqueous phase was transferred to 1.5 mL microcentrifuge tube. Next, 500 μL isoamyl alcohol was added in the tube, which was incubated at room temperature for 10 min and centrifuged at 12,000 × *g* for 5 min at 4 °C. The pellet was washed using 1 mL 70% ethanol, and suspended in 50 μL H_2_O.

For the plant samples, leaves or roots (cut to be a diameter of about 3–4 cm) were frozen and powdered, and nucleic acid was extracted based on the protocols used for fungal samples.

### Rapid nucleic acid extraction using quartz sand

Leaf samples (3–4 cm) were placed in 2-mL microcentrifuge tubes with 0.5 g quartz sand (8–16 meshes). The samples were ground using a plastic pestle in the presence of 100 μL extraction buffer (1 μL TCEP (100 mM), 1 μL Tris (2-carboxyethyl) phosphine (0.5 mM), 1 μL RNA Carrier and 97 μL H_2_O) for 5 min.

### Rapid nucleic acid extraction using MN patch

MN was synthesized according to a published protocol^38^. Briefly, an MN mold was first cleared in an ultrasonic bath for 5 min. Next, 1 mL 10% polyvinyl alcohol (PVA) solution was added to the mold, which was placed in a sealed vacuum chamber (600 mm Hg) for 20 min to draw the PVA solution into the needle cavities. The mold was then kept in the vacuum at 25 °C for 24 h. The MN patch contained an 11 × 11 microneedle array. The height and base of the needle were 600 µm and 300 µm, respectively. The spacing tip to tip was 600 µm.

For the MN patch-based nucleic acid extraction, and a MN patch was placed on a leaf and pressed gently by hand for 10 s. The MN patch was peeled off and collected in 50 μL H_2_O for further analysis.

### Pathogenic RNA detection in solution

A 2.5-μL sample was added to 10 μL prepared DProbe, and incubated at room temperature for 10 min. Then, 10 μL urease (10 nM, cat. no. U0017, EC 2.5.1.5; TCI, Tokyo, Japan), and 77.5 μL colorimetric mixture (10 μL phenol red (2.5 mM) ordered from Innochem (Beijing, China), 10 μL urea (5M, CON_2_H_4_) and 55.5 μL H_2_O) were added. The mixture was measured by a Synergy H1 microplate reader to record the absorbance at 560 nm, and was also photographed by a HUAWEI P40 smartphone.

### Preparation of origami papers

Filter paper was cut into A4 size (297 cm × 210 cm). A wax printer (Xerox ColourQube 8580/8880 N) was utilized to print the filter paper. The printed paper was heated at 170 °C for 10 s using a hot plate to melt the wax. The melted wax diffused through the paper, thus form hydrophobic pattern of hydrophilic reaction regions (3 mm in diameter). The reagents containing DProbe (5 μL, 100 nM), urease (10 nM) in 6 nM pullulan ((cat. no. A60187) purchased from Innochem (Beijing, China)), and colorimetric mixture (5 μL (1 μL phenol red (1.25 mM), 1 μL urea (2.5 M) and 3 μL H_2_O)) were dropped on each page of the printed paper to finish the preparation of the origami paper.

### Origami paper-based detection

A 5-μL sample was loaded on Page 1 through the upper window of a smartphone attachment. The origami paper was then moved to the bottom of the chamber. Folding processes for the origami paper are achieved via control sticks (Page 2 to Page 1, Page 3 to Page 2, and Page 4 to Page 3). Origami papers were photographed via a smartphone, and the images were analysed using a smartphone app (termed *ifDiag*).

### Histological observation and biomass measurement of *Pst* in infected wheat samples

First, the inoculated wheat leaf was decolourized and made transparent using 95% ethanol and chloral hydrate^28^. The samples were then treated in KOH (1 M) at 121 °C for 5 min and stained with 20 μg/mL wheat germ agglutinin Alexa-488 (Invitrogen) for 15 min. The samples were observed by fluorescence microscopy (Olympus BX63, excitation wavelength 450–480 nm, emission wavelength 515 nm).

Fungal biomass measurement was based on qPCR, using total DNA extracted from infected wheat leaves at different day post inoculation. The ratio of total fungal DNA to total wheat DNA was assessed by normalizing the data to the wheat gene TaEF-1α and the *Pst* gene PstEF1^50^. The primers used for detecting TaEF-1α and PstEF1 are listed in Supplementary Table 5.

### Gel electrophoresis

Gel electrophoresis was carried using 5% agarose gel stained with 1 × Gelred (Biotium, cat. no. 41001), and then was performed in 1 × TAE buffer at 150 V for about 60 min. After electrophoresis, the gel was visualized via the Gel Doc XR + system (BioRad, USA).

### qPCR and RT-qPCR detection

qPCR was performed to analyse fungal DNA samples using the Platinum SYBR Green qPCR SuperMix-UDG (cat. no. 11744100, Thermo) on the CFX96 Thermal Cyclers (Bio-Rad). qPCR conditions were as follows: 50 °C for 5 min, 94 °C for 5 min, and then 45 cycles of 94 °C for 15 s, 60 °C for 15 s and 72 °C for 45 s. The qPCR mixture contained 2.5 μL fungal DNA sample, 10 μL of SYBR Green qPCR Supermix, 2 μL primers (1 μL each of 10 mM forward and reverse primer) and 5.5 μL H_2_O. Expression levels of pathogenesis-related genes were measured using RT-qPCR. Total RNA (2 μg) was used for reverse transcription with a RevertAid First Strand cDNA Synthesis Kit (MNI, K1622). Diluted cDNA (1:5; 2 μL) was utilized in the following qPCR procedure. The expression levels of all tested genes were normalized to PstEF1. qPCR primers are listed in Supplementary Table 5.

### Statistics and reproducibility

Figures were created using Origin 2019. Two-sided Welch’s *t* test was used for all statistical comparisons and calculated by SPSS 25. The reproducibility of the results was assessed using a minimum of three independent experiments except Figs. 1c, 3a, Supplementary Fig. 31f, and Supplementary Fig. 36b, which were conducted once. “n” in the legend indicates the number of independent experiments. Receiver operating characteristic (ROC) curves were generated using GraphPad Prism 8.

### Reporting summary

Further information on research design is available in the Nature Portfolio Reporting Summary linked to this article.

### Supplementary information

Supplementary Information

Reporting Summary

### Source data

Source Data

## Data Availability

All data generated or analyzed during this study are included in this article and its Supplementary Information file. Source data are provided with this paper.

## Code availability

The code and detailed instructions for the in-field diagnostics crop diseases (ifDiag) app can be available on GitHub at https://github.com/Nelson233/Origami.
