# Supplementary material for: Precise in-field molecular diagnostics of crop diseases by smartphone-based mutation-resolved pathogenic RNA analysis
Source: Nat Commun. 2023 Jul 19;14:4327. doi: 10.1038/s41467-023-39952-x (PMC10356797; doi:10.1038/s41467-023-39952-x)
Supplement: Supplementary file 1 — Supplementary Information [file 41467_2023_39952_MOESM1_ESM.pdf]

Precise in-field molecular diagnostics of crop diseases by smartphone-based mutation-resolved pathogenic RNA analysis

Zhang *et al.*

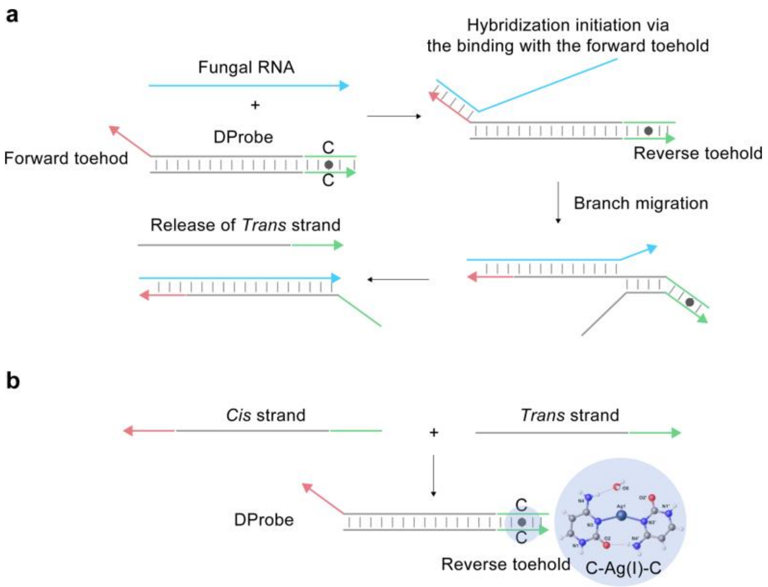

Supplementary Fig. 1. Illustration of the TMSD reaction and cytosine-Ag(I)-cytosine artificial base pair.

a, TMSD reaction. In the DProbe, the *Cis* strand hybridizes with a shorter sequence (the *Trans* strand), yielding a single-stranded overhang domain (in pink). The fungal RNA binds with the overhang domain, forming a "toehold" to force the hybridization with the *Cis* strand[1,2]. The sequential hybridization of the fungal RNA and the *Cis* strand along with the breakage of the DProbe hybrid yields branch migration. Eventually, the *Trans* strand is bound only with a short domain (reverse toehold) in the *Cis* strand that is not complementary to the fungal RNA, and disengages from the *Cis* strand. The net effect of the strand displacement process is determined by the formation of new base pairs within the forward toehold and the disruption of the former base pairs within the reverse toehold. By tuning the toehold domain in the DProbe, TMSD allows to yield hindrance for non-target RNA binding and permission of target RNA binding based on their different affinity towards the *Cis* strand. **b**, Cytosine-Ag(I)-cytosine artificial base pair. Ag(I) ion binds between the N3 nitrogen atoms of cytosine in the cytosine–cytosine mismatch pairs, forming cytosine-Ag(I)-cytosine (C-Ag(I)-C) artificial base pair[3]. By designing a C-C mismatch in the reverse toehold region, Ag(I) ion allows to insert into the DProbe via forming the artificial base pair.

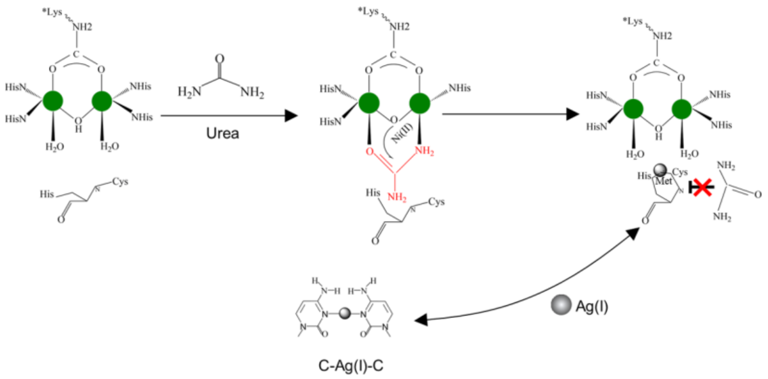

Supplementary Fig. 2. Urease inhibition by Ag(I) ion.

Urease is an enzyme that catalyzes the hydrolysis of urea to yield ammonia, which leads to a pH increase. Ag(I) ion exhibits strong inhibition effect on urease by binding the His-Cys-Met site of urease[4]. Ag (I) ion can be freed from C-Ag(I)-C artificial base pair.

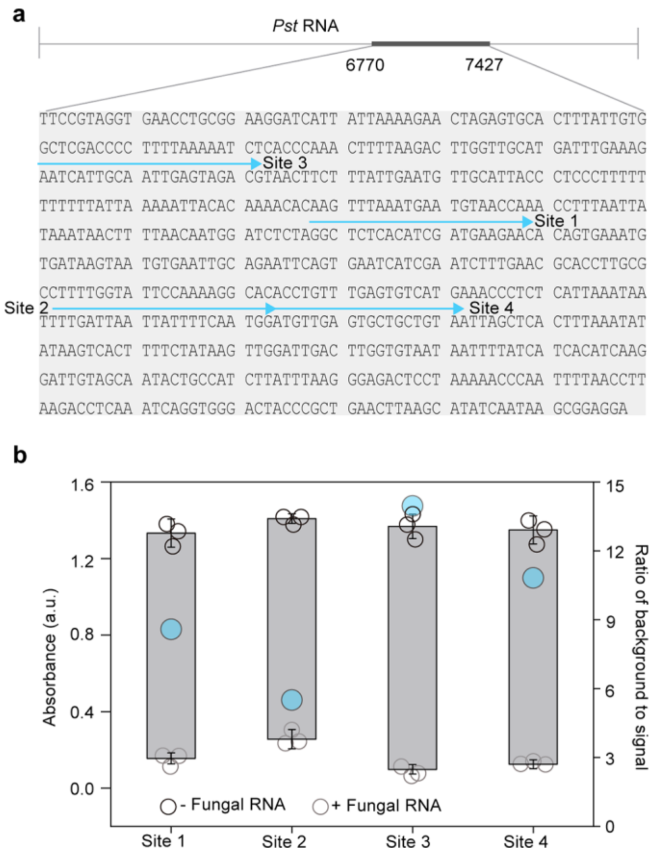

Supplementary Fig. 3. Visual detection of RNA fragments on ITS of *Pst*.

a, Four RNA fragments with 22-nt length on ITS of *Pst* were chosen as the target sites. **b,** Absorbance response of the assay by choosing the 4 fragments as the target sites. Absorbance was measured at the wavelength of 560 nm. Concentrations of RNA fragment, DProbe, urease, urea and phenol red were 200 nM, 100 nM, 1 nM, 500 mM and 250 µM. Data in **b** are means ± SD (n = 3). Based on the ratio of background to signal absorbance, the binding site 3 (6831-6852) in the ITS of *Pst* yielded a highest colour change using the assay, thus was chosen as the target RNA fragment. Source data are provided as a Source Data file.

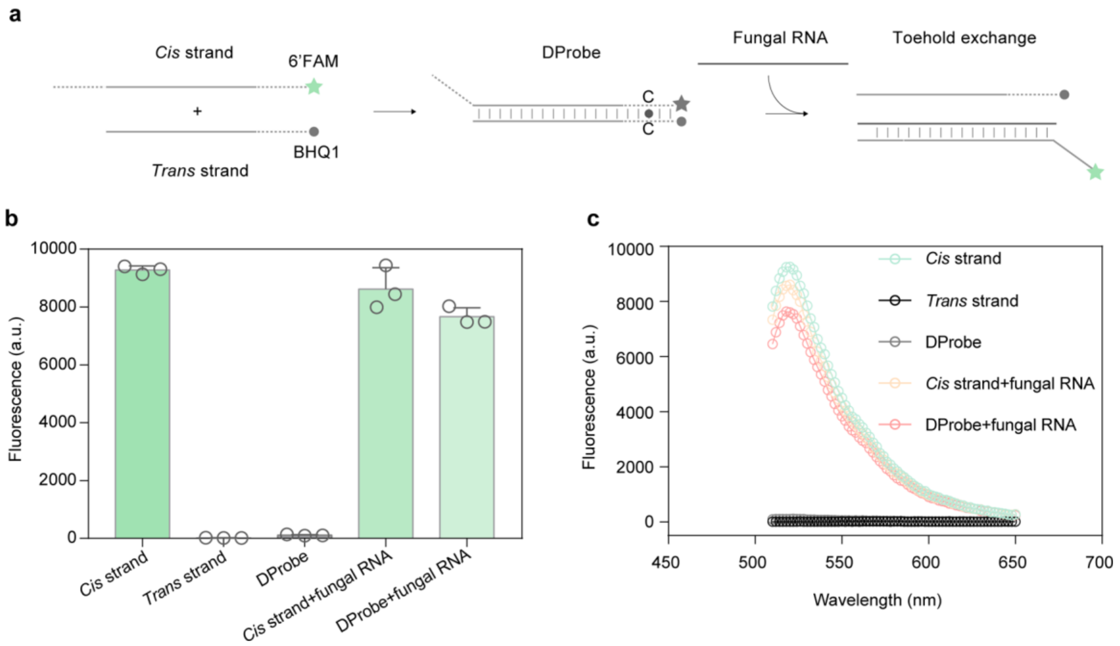

Supplementary Fig. 4. Investigation of the TMSD reaction via fluorescence analysis.

a, Scheme of the TMSD using labeled DProbes. Fluorophore, 6-Carboxyfluorescein (FAM) and quencher, Black Hole Quencher-1 (BHQ1) were modified at 3' end of the *Cis* strand and 5' end of the *Trans* strand, respectively. The formation of the DProbe via hybridizing the *Cis* strand and the *Trans* strand, leads to the proximity of FAM and BHQ1 and the quenching of FAM. The binding of the fungal RNA with the forward toehold on the DProbe initiates the TMSD reaction, and releases the *Trans* strand in the DProbe, restoring the fluorescence of FAM labeled in the *Cis* strand. **b-c**, Fluorescence intensity at 520 nm (**b**) and spectra at 480 nm excitation (**c**) of each hybrid involved in the TMSD reaction. Concentrations of the *Cis* strand, the *Trans* strand and the fungal RNA were 400 nM, 400 nM, and 600 nM. Data in **b** are means ± SD (n = 3). The single-stranded *Cis* strand (400 nM) yielded a fluorescence of 9281, and being quenched with the binding of the *Trans* strand, which indicates the formation of the double-stranded DProbe. The presence of the fungal RNA sequence turned on the fluorescence of the DProbe close to that of fungal RNA-*Cis* strand hybrid, indicating the efficient strand displacement reaction induced by the fungal RNA. Source data are provided as a Source Data file.

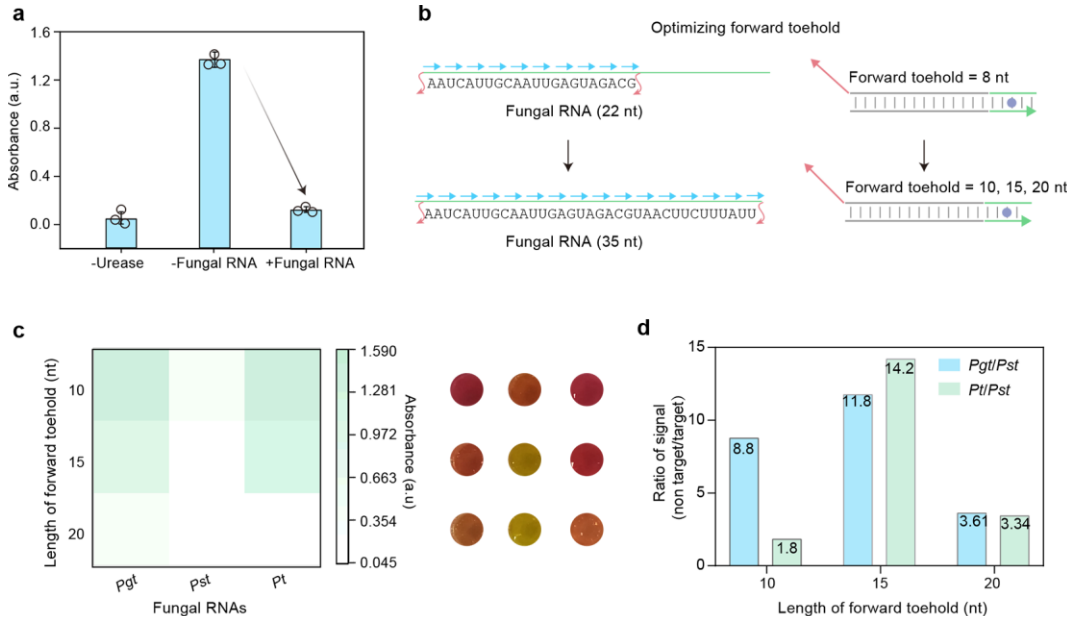

Supplementary Fig. 5. Optimization of the DProbe for detecting *Pst*.

a, Absorbance response towards long fungal RNAs extracted from *Pst* using the DProbe with a forward toehold of 8 nt and a reverse toehold of 7 nt. **b,** Forward toehold optimization from 8 nt to 10, 15 and 20 nt. **c,** Absorbance (left) and visual results (right) for the test of *Puccinia graminis* (*Pgt*), *Pst*, *Puccinia triticina* (*Pt*) using DProbes with different forward toeholds (10, 15 and 20-nt length) and a fixed reverse toehold (7 nt) designed based on the ITS sequence of *Pst*. **d,** Signal response for non-target RNA (from *Pgt* and *Pt*) to target RNA (from *Pst*). Absorbance was measured at the wavelength of 560 nm. Data in **a** are means ± SD (n = 3). The presence of the long fungal RNA did not sustain a colour change of phenol red to be that in the initial pH using the DProbe with a forward toehold of 8 nt and a reverse toehold of 7 nt (Supplementary Fig. 5a), indicating an insufficient TMSD reaction. This maybe because the long fungal RNA possesses complex secondary structures that hinder its hybridization with the DProbe, thus the TMSD reaction needs to be driven by a longer forward toehold. Meanwhile, to maintain the capacity of the assay to discriminate different fungal pathogens, we tested the assay response to *Pgt*, *Pt* and *Pst*. Highest ratio of signal response for non-target RNA (from *Pgt* and *Pt*) to target RNA (from *Pst*) was achieved using the DProbe with a 15-nt forward toehold and a 7-nt reverse toehold (Supplementary Fig. 5c and d). Therefore, the DProbe with a 15-nt forward toehold and a 7-nt reverse toehold was used as the optimized probe for detecting *Pst*. Source data are provided as a Source Data file.

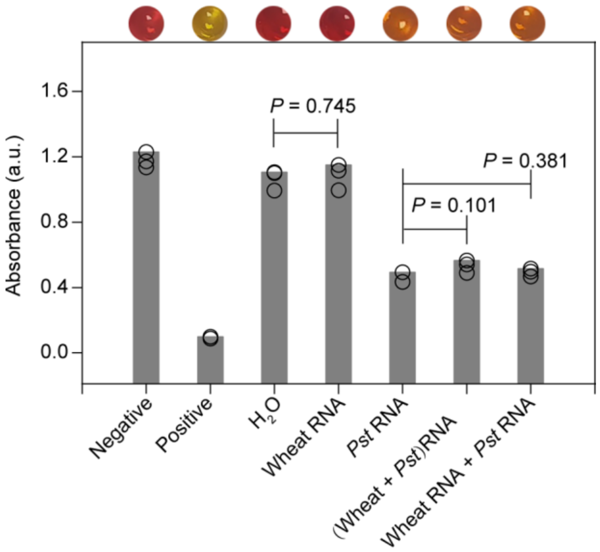

Supplementary Fig. 6. Investigation on the effect of wheat leaf RNAs on the detection of *Pst*. Absorbance (bottom) and visual results (upper) of the detection of *Pst* with the addition of wheat RNAs (labeled as "Wheat RNA + *Pst* RNA"), in the extraction of *Pst* infected wheat leaves (labeled as "(Wheat + *Pst*)RNA" and their controls. For the sample "Wheat RNA + *Pst* RNA", 10 mg *Pst* spores were inoculated onto cut wheat leaves with a square area of 2×2 cm (about 20 mg), and the wheat leave loaded with *Pst* spores was used for RNA extraction using Trizol reagent. For the sample "Wheat RNA + *Pst* RNA", RNA extracted from 20 mg wheat leaves was added into RNA extracted from 10 mg *Pst* spores. Concentrations of DProbe, urease, urea, and phenol red were 100 nM, 1 nM, 500 mM and 250 µM, respectively. Data are means ± SD (n = 3). Welch's two-sided unpaired t-test determined statistical significance. Source data are provided as a Source Data file.

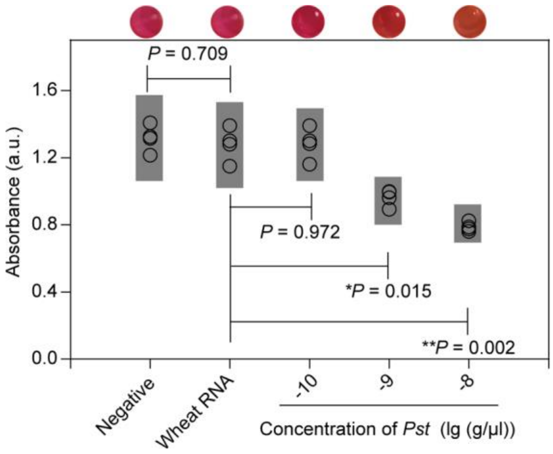

Supplementary Fig. 7. Estimation of the sensitivity of the assay for detecting *Pst* in wheat RNA matrix.

RNA extracted from 20 mg wheat leaves was added into RNA extracted from different amount of *Pst* spores. Absorbance (bottom) and visual results (upper) of the detection of *Pst* with concentrations ranged from 10^{-8} to 10^{-10} g/μL. Concentrations of DProbe, urease, urea and phenol red were 100 nM, 1 nM, 500 mM and 250 μM, respectively. Data are means ± SD (n = 4). Welch's two-sided unpaired t-test determined statistical significance: $*P < 0.05$, $**P < 0.01$. The assay allowed to detect 1.0 ng/μL *Pst* in the presence of wheat RNA matrix. Source data are provided as a Source Data file.

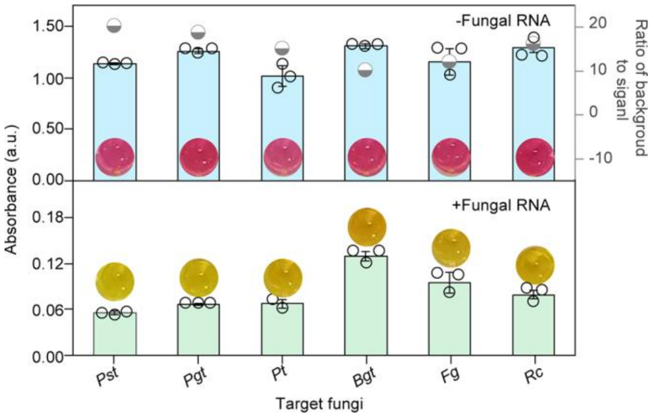

Supplementary Fig. 8. Visual detection of six wheat pathogenic fungi.

Fungal RNA was detected using the DProbe with a 15-nt forward toehold, and a 7-nt reverse toehold. Absorbance was measured at the wavelength of 560 nm. Data are means ± SD (n = 3). The ratio of background to signal of each fungus was over 10. Concentrations of DProbe, urease, urea, and phenol red were 100 nM, 1 nM, 500 mM and 250 μM, respectively. Source data are provided as a Source Data file.

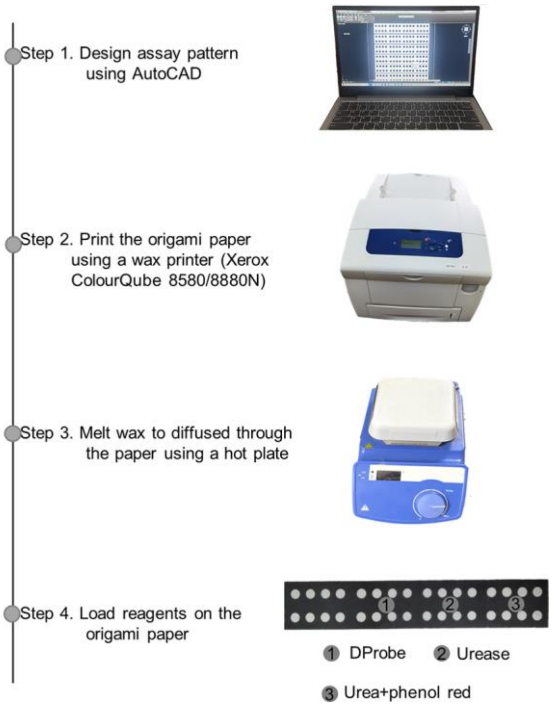

Supplementary Fig. 9. Illustration of the preparation of origami papers.

Layout of origami papers is designed using AutoCAD2019. Based on the design, the origami papers are printed using a wax printer, Xerox8580N, and heated using a heating plate to create detection spots that surrounded by a hydrophobic barrier based on the reported protocol[5]. The origami papers are designed with 4 sectors, and separately loaded with sample, DProbe, urease and urea/phenol red.

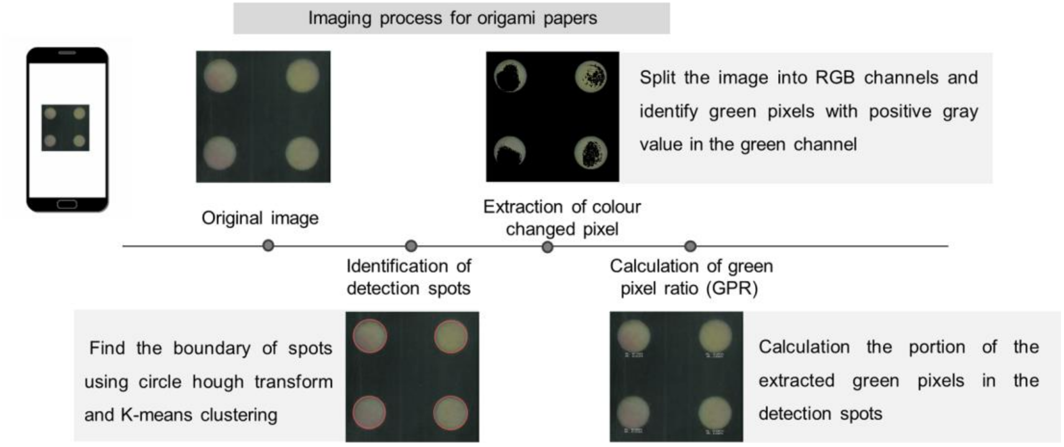

Supplementary Fig. 10. Illustration of image processing for origami papers.

Circle segment as the detection spots was identified using Circle Hough Transform and K-means clustering that can distinguish the coloured zoom from the black background[6]. To alleviate the effect of the colour nonuniformity on the quantification of the colour change, we designed an algorithm that extract the number of the colour changed pixels within the circular detection region, rather than the average gray values. The number of the colour changed pixels within the detection region can digitally indicate the reaction of urease and its activity. Specifically, the pixels within the circular detection region were all separated into RGB channels and recorded with gray values in each channel. Based on the principle of the assay, the inhibition of urease reduces the production of ammonia, in turn reduces the pH of the solution and turn the phenol red to be yellow. And compared to the red colour which phenol red turned to be in a raised pH, the pixel in yellow colour can yield a positive gray value in the green channel. Therefore, we designed the algorithm to record the number of the pixels that have a positive gray value in the green channel, and defined the ratio of the number of pixels with positive gray value in the green channel to the number of all the pixels within the circular detection region as the green pixel ratio (GPR) value. The GPR value is used to estimate the activity of urease, and indicate the presence of the fungal RNA.

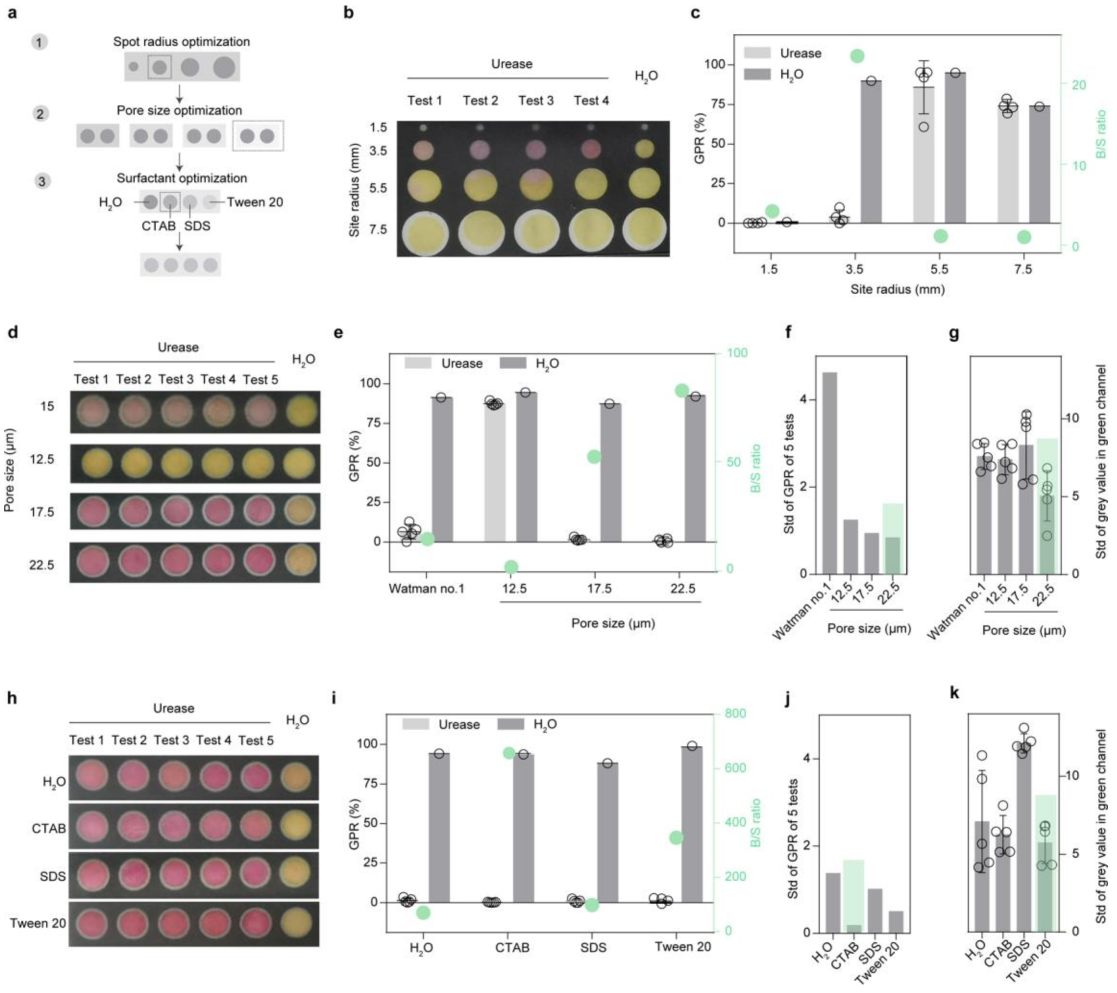

Supplementary Fig. 11. Optimization of origami papers for colorimetric detection.

a, Illustration the optimization process. **b**, **d**, **h**, Photos of origami papers in the presence and absence of urease in different paper conditions: detection spots with different diameters of (**b**), papers with different pore sizes (**d**) and with the modification using different surfactant (**h**). **c**, **e**, **i** GPR values of origami papers in the presence and absence of urease and its ratio tested in **b, d, h**. **f**, **j**, Standard deviation (Std) of GPR value obtained from the replicated tests in **d** and **h**, respectively. **g**, **k**, Std of gray value of each pixel in the green channel within the circular detection zone tested in **d** and **h**, respectively. B/S ratio: ratio of GPRs in the absence and presence of urease. Uniformity of color change signal is estimated as Std of gray value of each pixel in green channel. Concentrations of urease, urea, and phenol red were 1 nM, 500 mM and 250 µM, respectively. Data in **e**, **i**, **g** and **k** are means ± SD (*n* = 5). Data in **c** are means ± SD (*n* = 4). Source data are provided as a Source Data file.

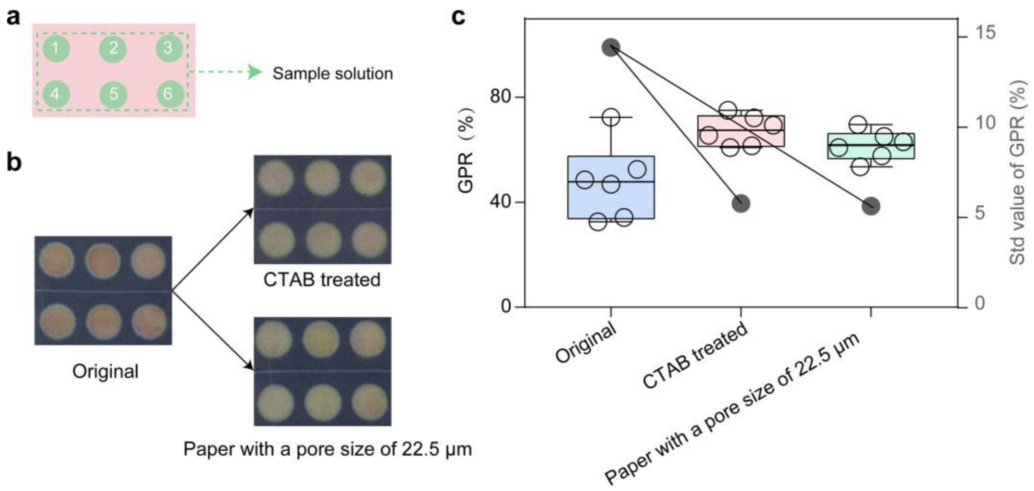

Supplementary Fig. 12. Investigation of the colour homogeneity for detecting *Pst* with the optimized origami papers.

a, Paper design for detecting *Pst*. **b**, Photographs of the origami papers for the test of *Pst*. **c,** Comparison of the GPR value and its standard deviation using original origami papers, CTAB-treated origami papers, origami papers with a pore size of 22.5 µm. The bottom and top lines of the box represent the first and third quartiles respectively, the middle line in the box indicate the median value. The whisker lines indicate the minimum and maximum value within 6 independent testes. Compared to the usage of original papers, the standard deviation of GPR values using the optimized origami papers is dramatically reduced (from 14.42 to 5.77 (the CTAB-treated origami paper), and 5.64 (the origami paper with a pore size of 22.5 µm)), indicating that the optimization of the pore size and the addition of surfactant yield an improved colour homogeneity. Source data are provided as a Source Data file.

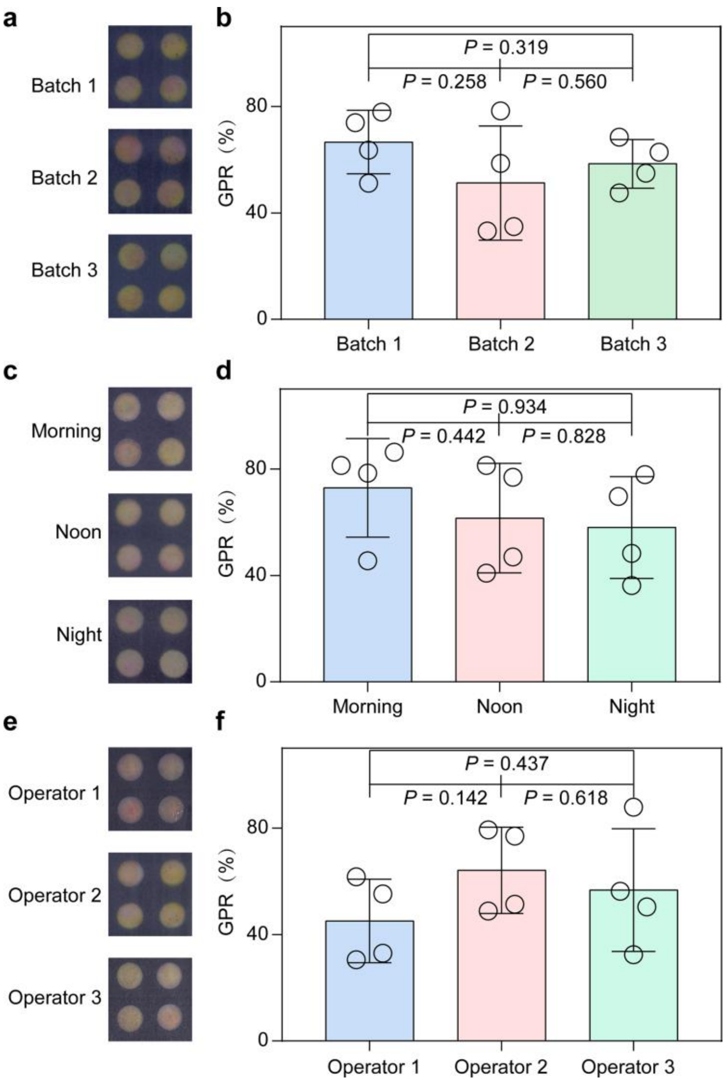

Supplementary Fig. 13. Detection of *Pst* using different batches of origami papers, at different operation times, and by three different operators.

(**a**, **c**, **e**) Photographs of the origami papers for the test using different batches of origami papers (**a**), at different operation times (**c**), and by three different operators (**e**). (**b**, **d**, **f**), GPR values of the origami papers for the test using different batches of origami papers (**b**), at different operation times (**d**), and by three different operators (**f**). Data in **b**, **d** and **f** are means ± SD (n = 4). P values are obtained via Welch's two-sided unpaired t-test. Source data are provided as a Source Data file.

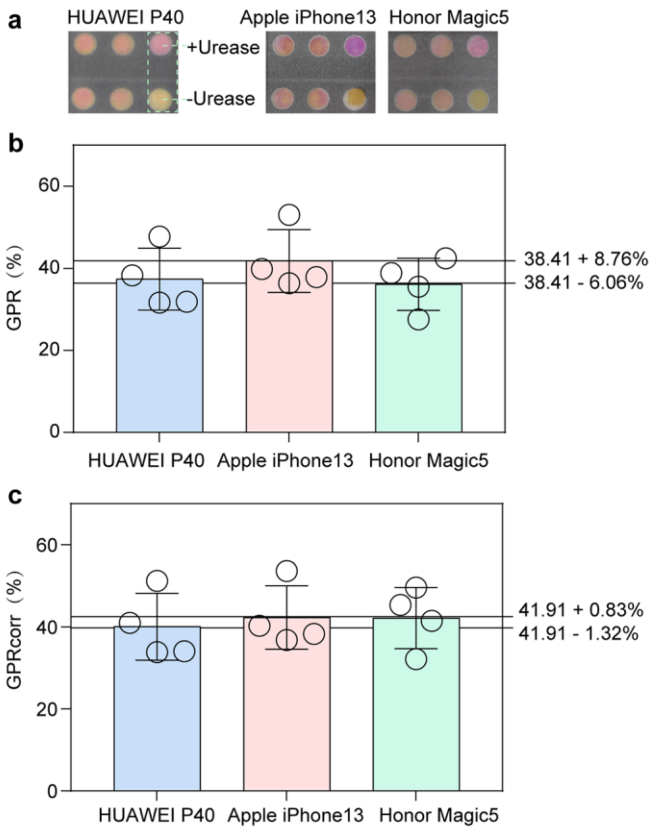

Supplementary Fig. 14. Demonstration of the colour referring strategy to record the colour readout using different smartphones.

a, Photographs of the origami papers for the tests using different smartphones. **B**, GPR values of the origami papers tested in **a**. **c**, GPR_{corr} values of the origami papers tested in **a**. GPR_{corr} = (GPR_{test} – $GPR_{+urease}$) / ($GPR_{-urease}$ - $GPR_{+urease}$), where GPR_{test} is the GPR value of the detection spot, $GPR_{+urease}$ is the GRP value of the spot in the presence of urease, urea and phenol red, and $GRP_{-urease}$ is the GRP value of the spot in the presence of urea and phenol red. Positive and negative deviation values were defined as ((GPR_{max} – GPR_{ave}) / GPR_{ave}) × 100, ((GPR_{ave}–GPR_{min}) / GPR_{ave}) × 100, respectively, and GPR_{max} GPR_{min}, and GPR_{ave} are the maximum, minimum, and mean values of GPR phoned by three cell phones. Data in **b** and **c** are means ± SD (*n* = 4). Source data are provided as a Source Data file.

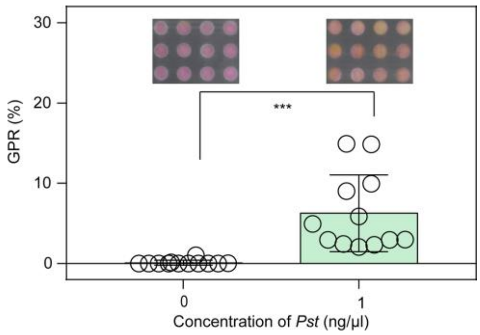

Supplementary Fig. 15. The test of low-level *Pst* using the origami papers.

Detection of the samples with 1 ng/μL *Pst* using the origami papers. Data are means ± SD. (n = 12). P values from Welch's two-sided unpaired t-test: ***$P < 0.001$. Source data are provided as a Source Data file.

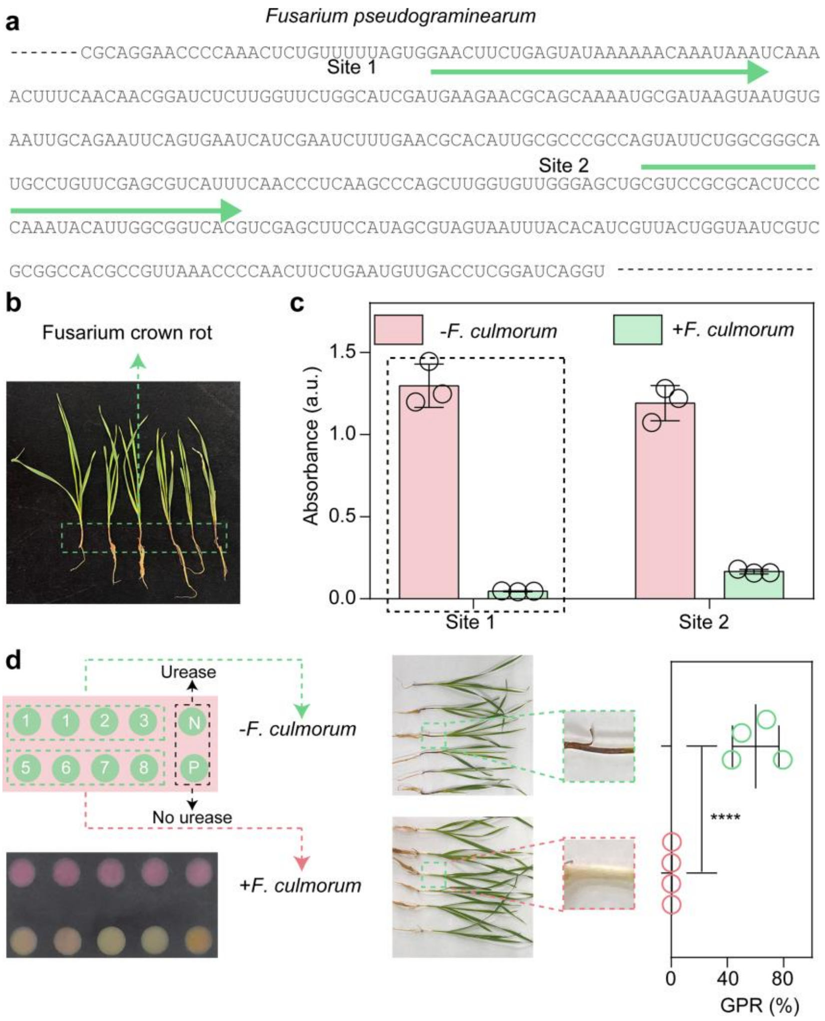

Supplementary Fig. 16. Visual detection of the infection of *Fusarium culmorum*.

a, Fragment of internal transcribed spacer 1 RNA of *Fusarium culmorum* (*F. culmorum*) to be detected. **b,** Phenotyping of wheat samples infected with *F. culmorum*. **c,** Absorbance at 560 nm of the test of the wheat root with or without *F. culmorum* infection. **d,** Design and the photographs of the origami paper for *F. culmorum* detection (left), phenotyping of wheat samples with or without the infection of *F. culmorum* (middle), and the GPR values of the origami papers testing in the presence and absence of *F. culmorum* (right). Data in **c** and **d** are means ± SD (**c**, $n = 3$; **d**, $n = 4$), P values from Welch's two-sided unpaired t-test in **d**: $****P < 0.0001$. Source data are provided as a Source Data file.

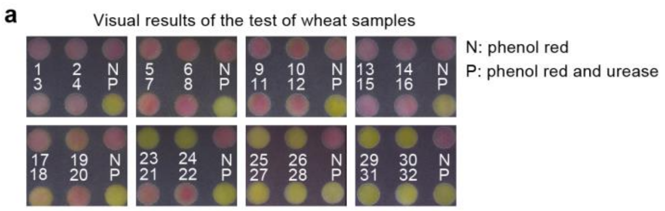

b GPR value of each wheat sample

(Rows: Sample number / GPR (%))

1	2	3	4	5	6	7	8
0.02	0.00	5.13	5.84	0.23	0.00	0.00	0.36
9	10	11	12	13	14	15	16
0.00	2.50	2.56	9.38	1.07	4.17	0.08	6.00
17	18	19	20	21	22	23	24
0.38	89.47	0.00	0.00	0.00	9.39	89.91	79.91
25	26	27	28	29	30	31	32
90.91	91.96	92.82	94.91	89.96	88.88	83.96	72.96

Supplementary Fig. 17. Detection of *Pst* infection in thirty-two wheat leaf samples using the colorimetric paper.

Visual test results (**a**) and GAR values (**b**) of wheat samples using the DProbe with a forward toehold of 15 nt and a reverse toehold of 7 nt. The results of eleven infected wheat leaves were shadowed in blue. Concentrations of DProbe, urease, urea, and phenol red were 100 nM, 1 nM, 500 mM and 250 μM.

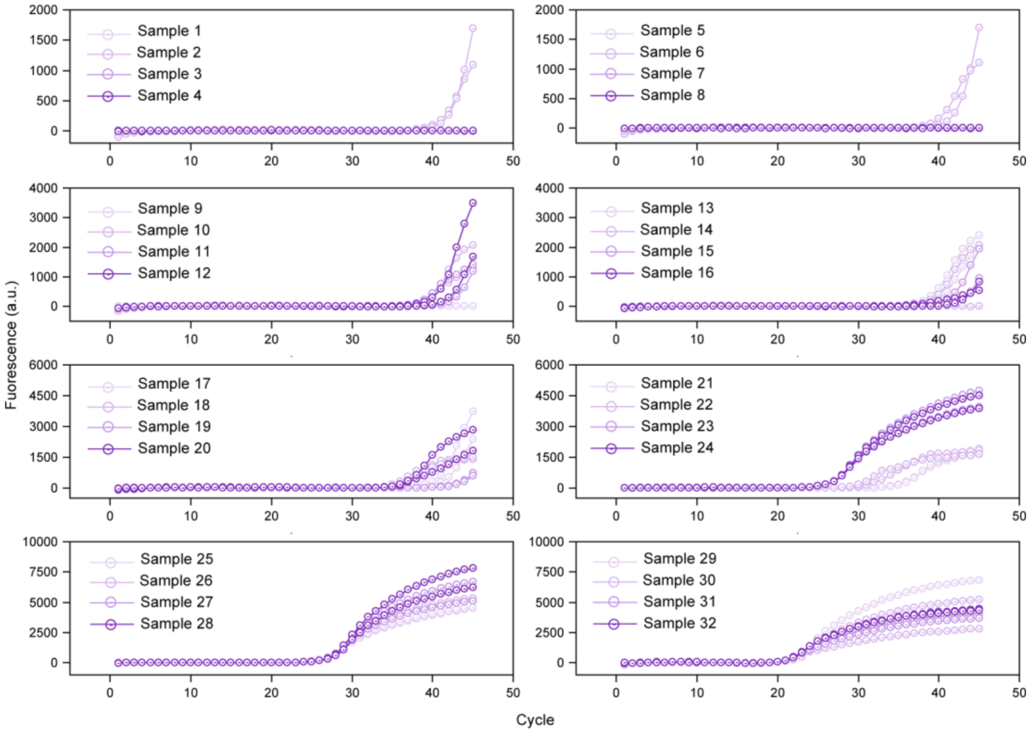

Supplementary Fig. 18. Detection of *Pst* infection in thirty-two wheat leaf samples using qPCR. Amplification curves of *Pst* DNA from the thirty-two wheat leaf leaves. Twenty-one samples were tested to be negative, and eleven samples were positive. Each sample was tested twice using qPCR method. Source data are provided as a Source Data file.

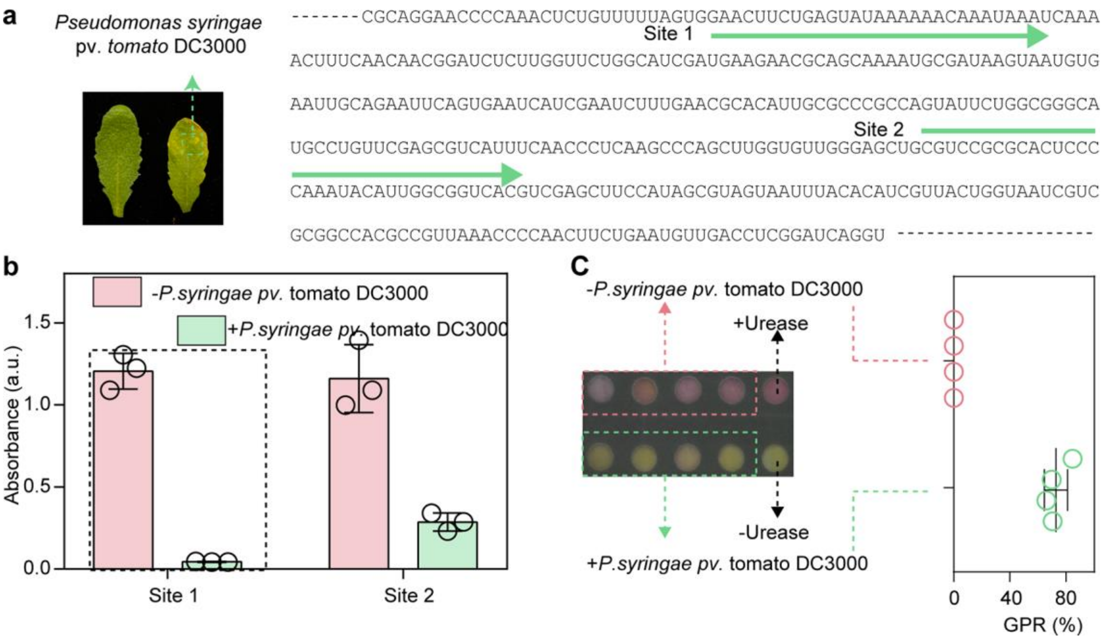

Supplementary Fig. 19. Visual detection of bacterial pathogen, *P. syringae pv. tomato* DC3000. **a,** Phenotyping of leaves of the Arabidopsis (left) and the fragment of 16S ribosomal RNA of *P. syringae* DC3000 to be detected. DProbes are designed to target the underlined sequences labeled as site 1 and 2. **b**, Absorbance at 560 nm of the test of the leaves of the Arabidopsis with or without the infection with *P. syringae pv. tomato* DC3000. **c**, Image and GPR values via testing the leaves of the Arabidopsis with or without the infection with *P. syringae pv. tomato* DC3000 using the DProbe targeting site 1. Data in **b** and **c** are means ± SD (**b**, $n = 3$; **c**, $n = 4$). Source data are provided as a Source Data file.

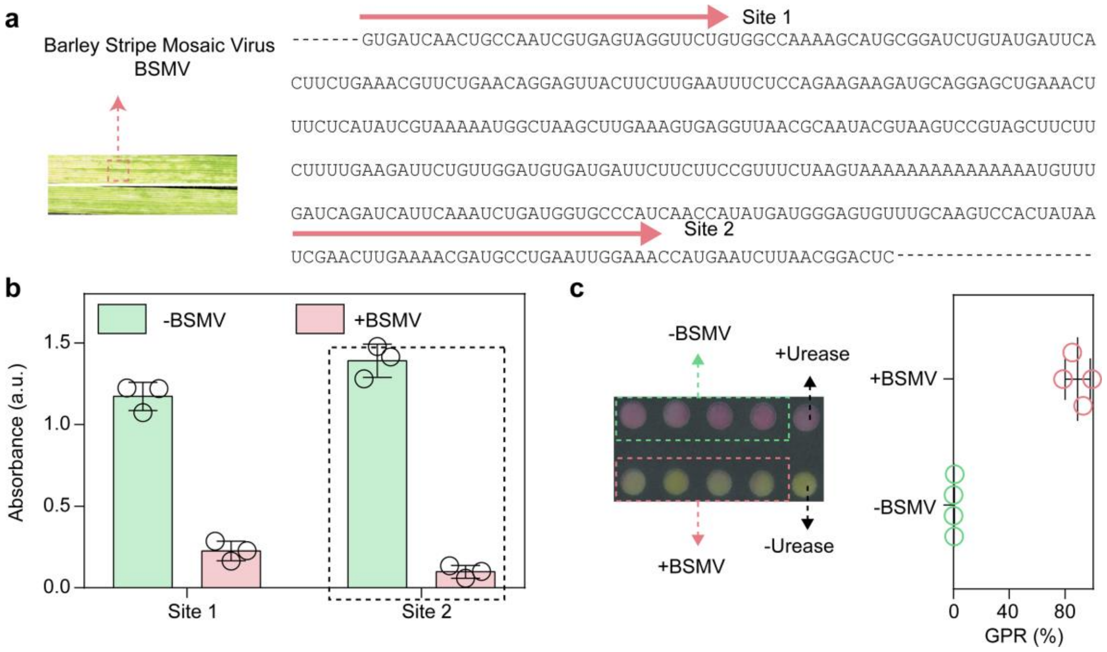

Supplementary Fig. 20. Visual detection of viral pathogen, BSMV.

a, Phenotyping of leaves of wheat (left) and the fragment of ND18 RNA gamma segment of BSMV to be detected. DProbes are designed to target the underlined sequences labeled as site 1 and 2. **b**, Absorbance at 560 nm of the test of the leaves of barley with or without the infection with BSMV using the DProbe targeting site 2. **c**, Image and GPR values via testing the leaves with or without the infection with BSMV. Data in **b** and **c** are means ± SD (**b**, *n* = 3; **c**, *n* = 4). Source data are provided as a Source Data file.

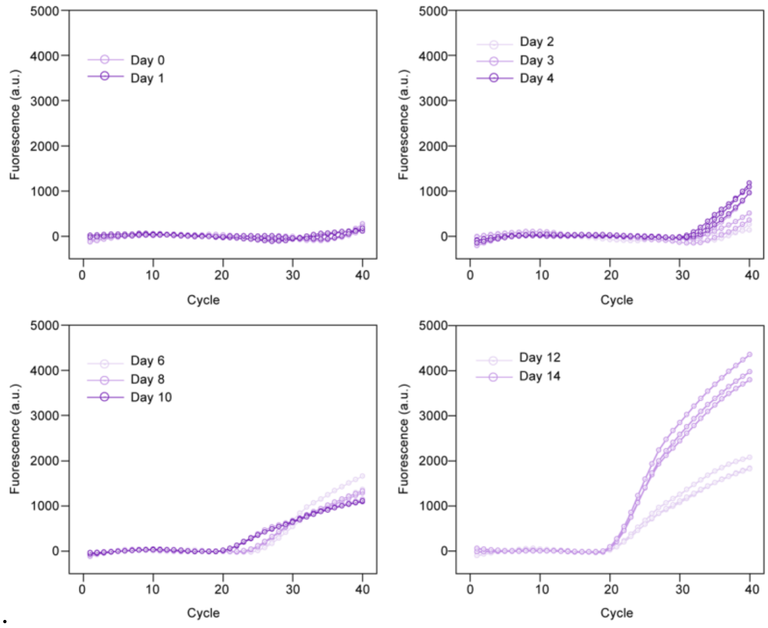

Supplementary Fig. 21. qPCR tests of *Pst*-infected wheat leaf samples with different inoculation time.

Amplification curves of fungal DNA extracted from the infected wheat leaves on day 0, 1, 2, 3, 4, 6, 8, 10, 12 and 14. Each leaf sample was tested by three times using qPCR method. Source data are provided as a Source Data file.

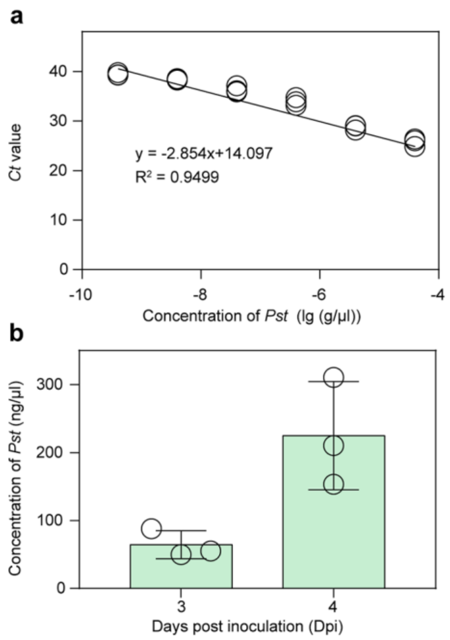

Supplementary Fig. 22. Estimation the detectable load of *Pst* in the infected wheat leaf samples using the colorimetric assay.

a, Linear relationship between the *Ct* value and the concentration of *Pst*. b, Concentrations of *Pst* in the 3-, and 4-day infected wheat leaves measured by qPCR. Data in **a**, **b** are means ± SD (n = 3). Source data are provided as a Source Data file.

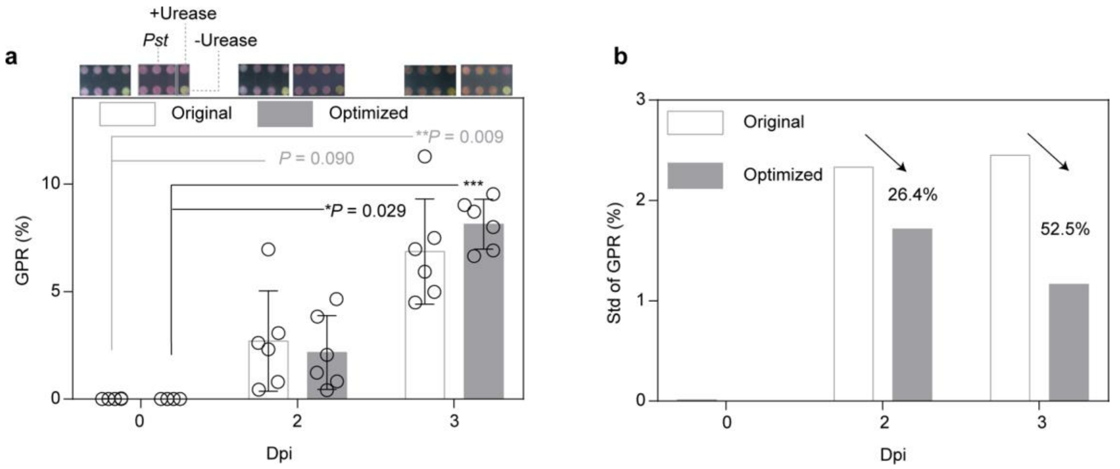

Supplementary Fig. 23. Comparison of the assay for detecting *Pst* infection on wheat leaves in day 0, 2 and 3 before and after the optimization of origami papers.

a, GPR values (below) and photos (upper) of the assay testing *Pst* infection on wheat samples in day 0, 2 and 3. **b**, Standard deviation (Std) of GPR value obtained from the replicated tests in **a**. Data in **a** are means ± SD (n = 6). Welch's two-sided unpaired t-test determined statistical significance in **a**: *$P < 0.05$, **$P < 0.01$, ***$P < 0.001$. The optimized papering condition for detecting *Pst* infection: paper with a pore size of 22.5 μm and CTAB loading. Concentrations of DProbe, urease, urea, and phenol red were 100 nM, 1 nM, 500 mM and 250 μM. Source data are provided as a Source Data file.

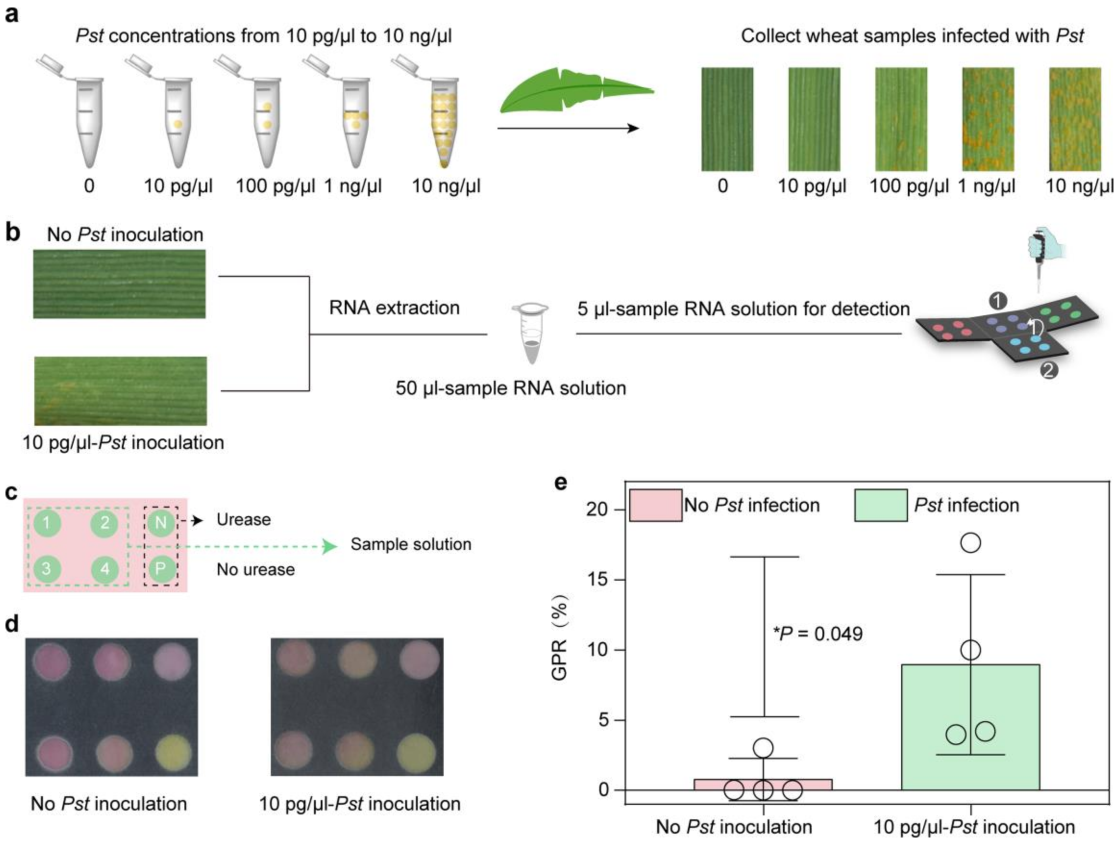

Supplementary Fig. 24. Detection of *Pst* in symptom-free infected wheat leaf samples using the colorimetric assay.

a, Phenotyping of the wheat samples inoculated with *Pst* at concentrations ranged from 10 pg/μL to 10 ng/μL. **b**, Scheme of *Pst* detection from the wheat leaves after 14 days of *Pst* inoculation using the origami papers. **c**, Design of the origami papers. **d**, Photographs of the origami papers for the test of *Pst* in the infected wheat leaves (inoculation dose, 10 pg/μL). **e**. GPR values of the test of non-infected wheat leaves and *Pst*-infected wheat leaves (inoculation dose: 10 pg/μL). Data in **e** are means ± SD (n = 4), P values from Welch's two-sided unpaired t-test in **e**: $*P < 0.05$. Source data are provided as a Source Data file.

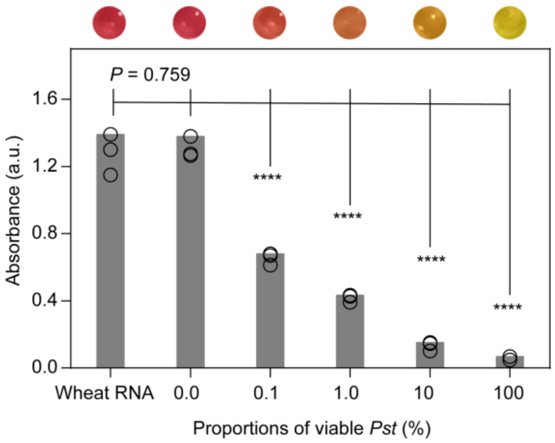

Supplementary Fig. 25. Estimation of the sensitivity of the assay for detecting viable *Pst* in wheat RNA matrix.

RNA extracted from 20 mg wheat leaves was added into RNA extracted from *Pst* mixtures. Absorbance (bottom) and visual results (upper) of the detection of *Pst* mixtures with 0%, 0.1%, 1%, 10% and 100% portion of viable spores. Concentrations of DProbe, urease, urea and phenol red were 100 nM, 1 nM, 500 mM and 250 µM, respectively. Upper: visual results of each reaction. Data are means ± SD ($n = 3$). Welch's two-sided unpaired t-test determined statistical significance: ****$P < 0.0001$. Source data are provided as a Source Data file.

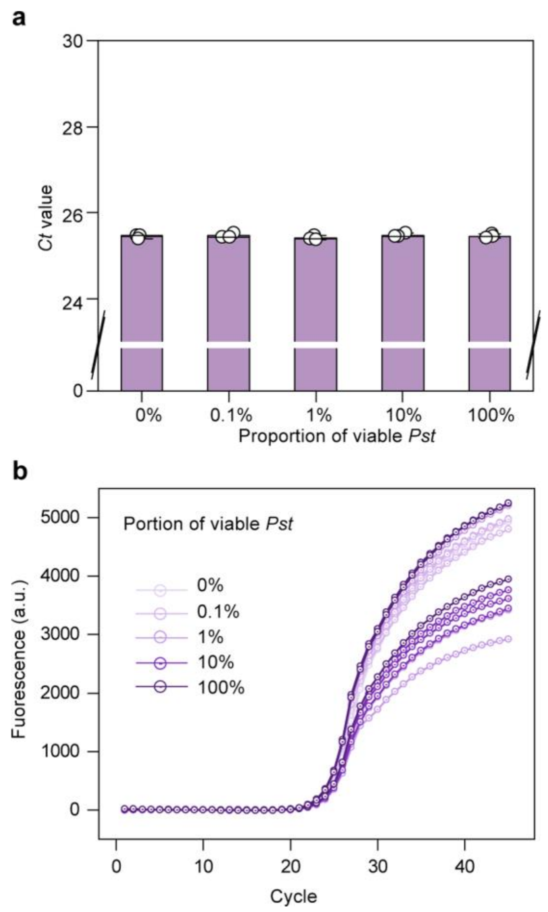

Supplementary Fig. 26. qPCR tests of *Pst* mixture with viable fungi and dead fungi.

a, *Ct* values of *Pst* mixture contained 0%, 0.1%, 1%, 10% and 100% viable fungi. **b**, Amplification curves of the mixed fungal samples in **a**. Each sample was tested three times using qPCR method. Data in **a** are means ± SD (*n* = 3). Source data are provided as a Source Data file.

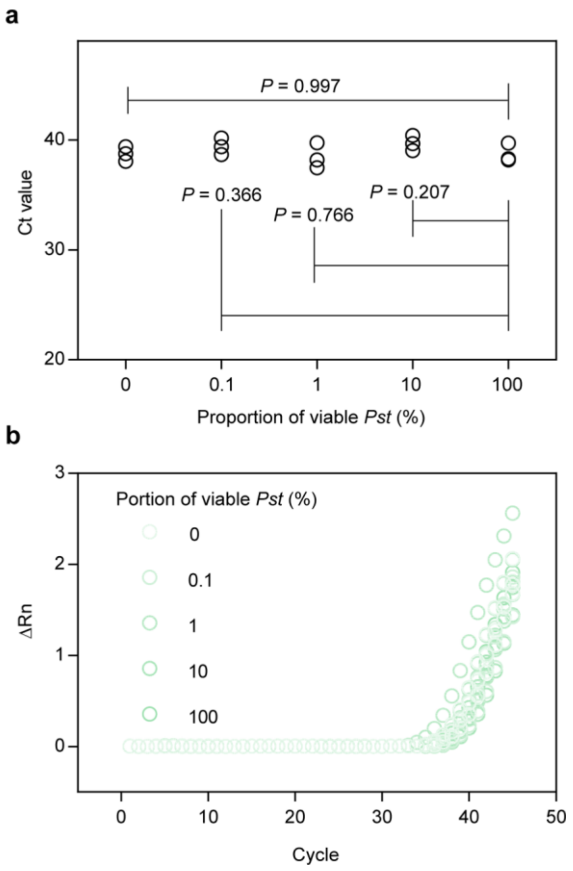

Supplementary Fig. 27. qPCR measurement of wheat infection using *Pst* mixture contained different portions of viable *Pst* at 0 day.

Ct values (**a**) and amplification curves (**b**) of *Pst* in wheat leaves under the infection of *Pst* mixtures with viable spores of 0%, 0.1%, 1%, 10%, and 100% at day 0. Each sample was tested three times using qPCR method. Data are means ($n = 3$). Welch's two-sided unpaired *t*-test determined statistical significance. Source data are provided as a Source Data file.

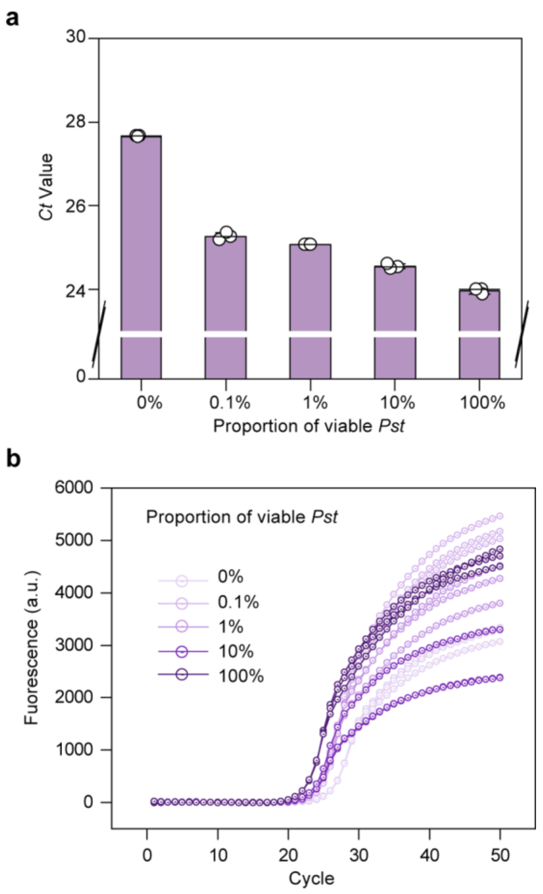

Supplementary Fig. 28. qPCR tests of wheat leaf samples inoculated with *Pst* mixed with different portions of viable fungi.

a, *Ct* values of wheat leaves inoculated with 0%, 0.1%, 1%, 10%, and 100% viable *Pst*. **b**, Amplification curves of the leaf samples in **a**. Each sample was tested three times using qPCR method. Data are means ± SD (n = 3). Source data are provided as a Source Data file.

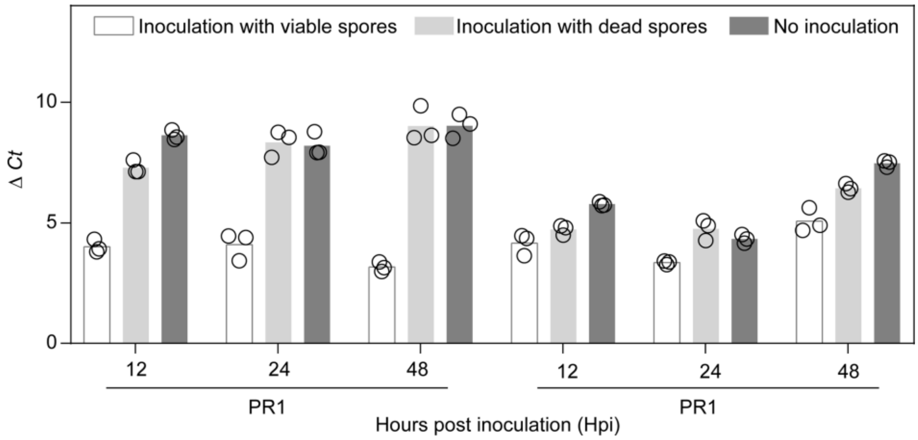

Supplementary Fig. 29. Expression of wheat pathogenesis-related genes in response to dead *Pst* spores.

The inoculated leaves with dead or viable *Pst* spores were harvested at 12, 24 and 48 h post inoculation. RT-qPCR was used to measure the expression level of the internal control gene *Pst* EF1-α and PR1, PR2. $\Delta Ct = Ct_{(\mathrm{PR1\ or\ PR2})} - Ct_{(\mathrm{PstEF1\text{-}\alpha})}$, where, $Ct_{(\mathrm{PR1\ or\ PR2})}$ is *Ct* values of PR1 or PR2 gene, $Ct_{(\mathrm{PR1\ or\ PR2})}$ is *Ct* values of *Pst* EF1-α gene. Data are means (n = 3). Source data are provided as a Source Data file.

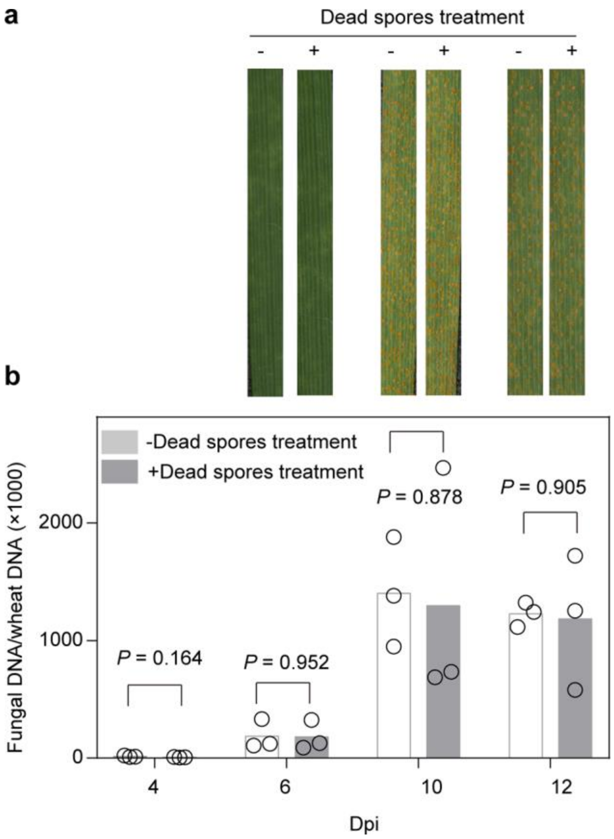

Supplementary Fig. 30. Effect of the pre-inoculation with dead spores on *Pst* infection. Phenotyping of wheat leaves (**a**) and *Pst* biomass (**b**) after 6 d-, 10 - and 12 d- infection of *Pst* with or without dead spore pre-inoculation. The fresh viable *Pst* spores were sequentially inoculated after two days of dead *Pst* spore inoculation. The inoculated leaves were harvested at 4-, 6-, 10-, and 12-day post inoculation after the second inoculation. Biomass of *Pst* between pre-treatment with dead spore and control group was measured by quantifying the wheat gene, TaEF-1α and the *Pst* gene, PstEF1. Data in **b** are means (n = 3). Welch's two-sided unpaired t-test determined statistical significance. Source data are provided as a Source Data file.

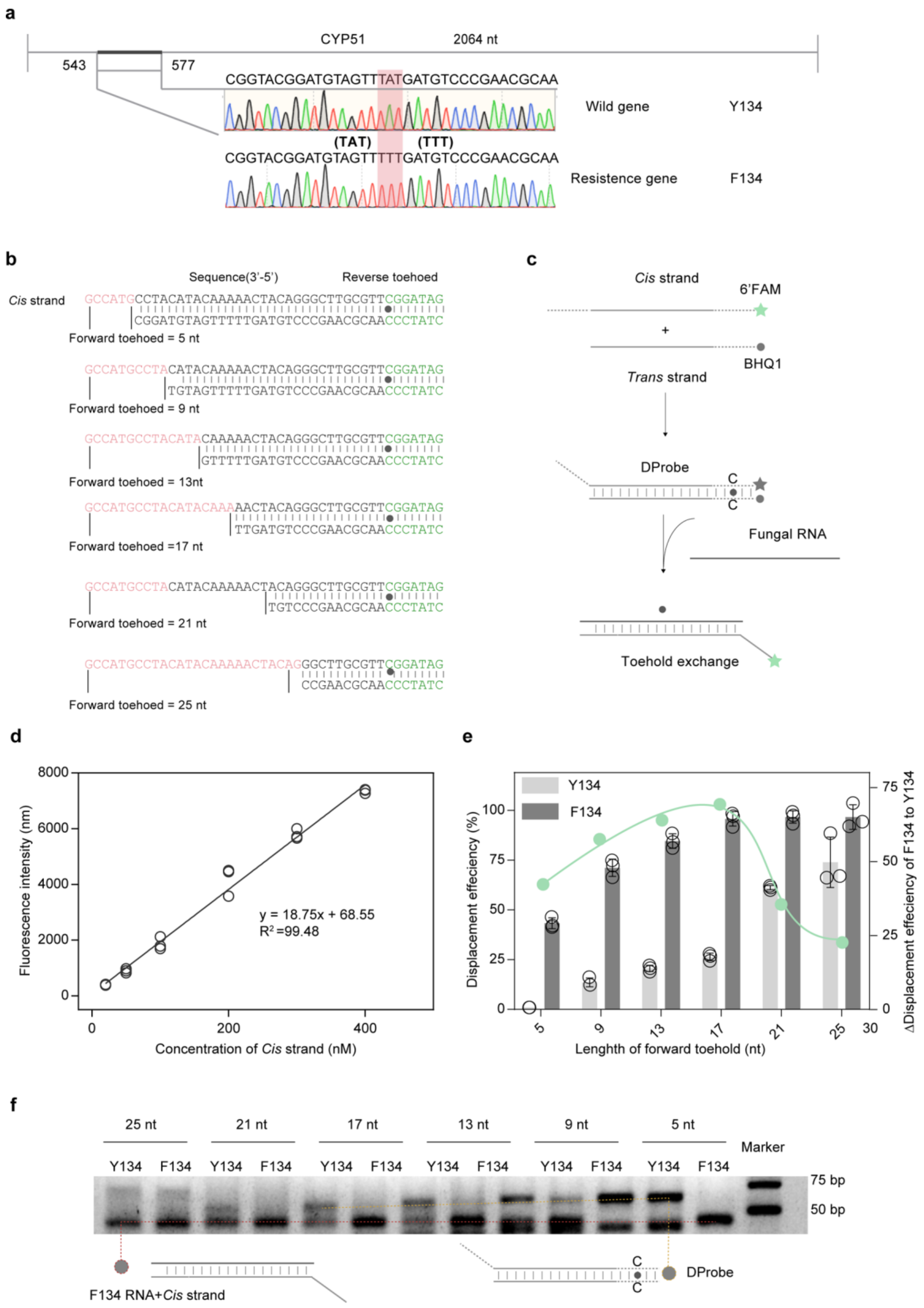

Supplementary Fig. 31. Optimization of DProbes for detecting Y134F mutation.

a, Sequencing result of mutation sites in *CYP51* gene. **b**, Design of DProbes with a tunable forward toehold ranging from 5 nt to 25 nt and a fixed reverse toehold of 7 nt. **c**, Scheme for the TMSD reaction using the labeled DProbe. **d**, Quantification of the concentration of the FAM-labeled *Cis*

strand. **e**, Measurement of the displacement efficiency towards Y134 RNA and F134 RNA using DProbes with different forward toehold lengths. **f**, Electrophoretic analysis of the TMSD reaction towards Y134 RNA and F134 RNA using DProbes with different forward toehold lengths. Fluorescence intensity in **d** and **f** was measured with an excitation wavelength of 480 nm and an emission wavelength of 520 nm. Concentrations of DProbe, urease, urea, and phenol red were 100 nM, 1 nM, 500 mM and 250 μM. The displacement efficiency was estimated as DE = ($Concentration_{\textit{Cis}\text{ in single strand}}$ / $Concentration_{\text{initial DProbe}}$)/100, where $Concentration_{\textit{Cis}\text{ in single strand}}$ is the concentration of the released *Cis* strand, $Concentration_{\text{initial DProbe}}$ is the concentration of DProbe used in the reaction. $Concentration_{\textit{Cis}\text{ in single strand}}$ was measured based on the standard curves used for quantification of FAM-labeled *Cis* strand, $Concentration_{\text{initial DProbe}}$ is 400 nM in the test. For electrophoretic analysis, DProbes were prepared with a 1.1:1 ratio of the *Trans* strand to the *Cis* strand, and annealed with a 400 nM concentration of DProbe. F134 RNA or Y134 RNA was added to achieve final concentrations of 600 nM RNAs and 400 nM DProbe. To distinguish DProbe from the hybrid of RNA and the *Cis* strand in the electrophoresis ladder, 30 nt poly-T was added to the 5' end of the *Trans* strand. Data in **d** and **e** are means ± SD (*n* = 3). Source data are provided as a Source Data file.

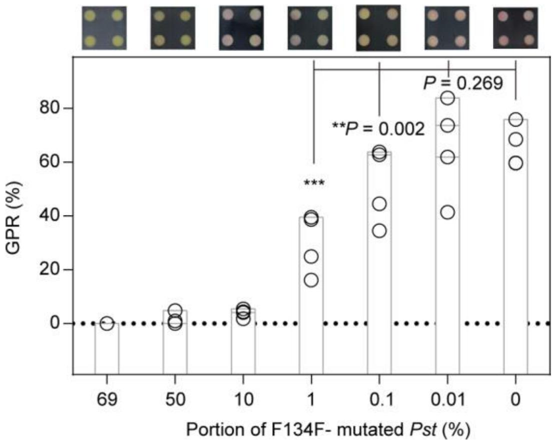

Supplementary Fig. 32. Detection of mutated dilution series in wheat RNA matrix.

RNA extracted from 20 mg wheat leaves was added into RNA extracted from *Pst* mixtures. Absorbance (bottom) and visual results (upper) of the detection of *Pst* mixtures containing wild and Y134F-mutated *Pst*, the portions of Y134F-mutated *Pst* are 69%, 50%, 10%, 1%, 0.1%, and 0.01%. Concentrations of DProbe, urease, urea and phenol red were 100 nM, 1 nM, 500 mM and 250 μM, respectively. Data are means ± SD (n = 4). Welch's two-sided unpaired t-test determined statistical significance: $**P < 0.01$, $***P < 0.001$. Source data are provided as a Source Data file.

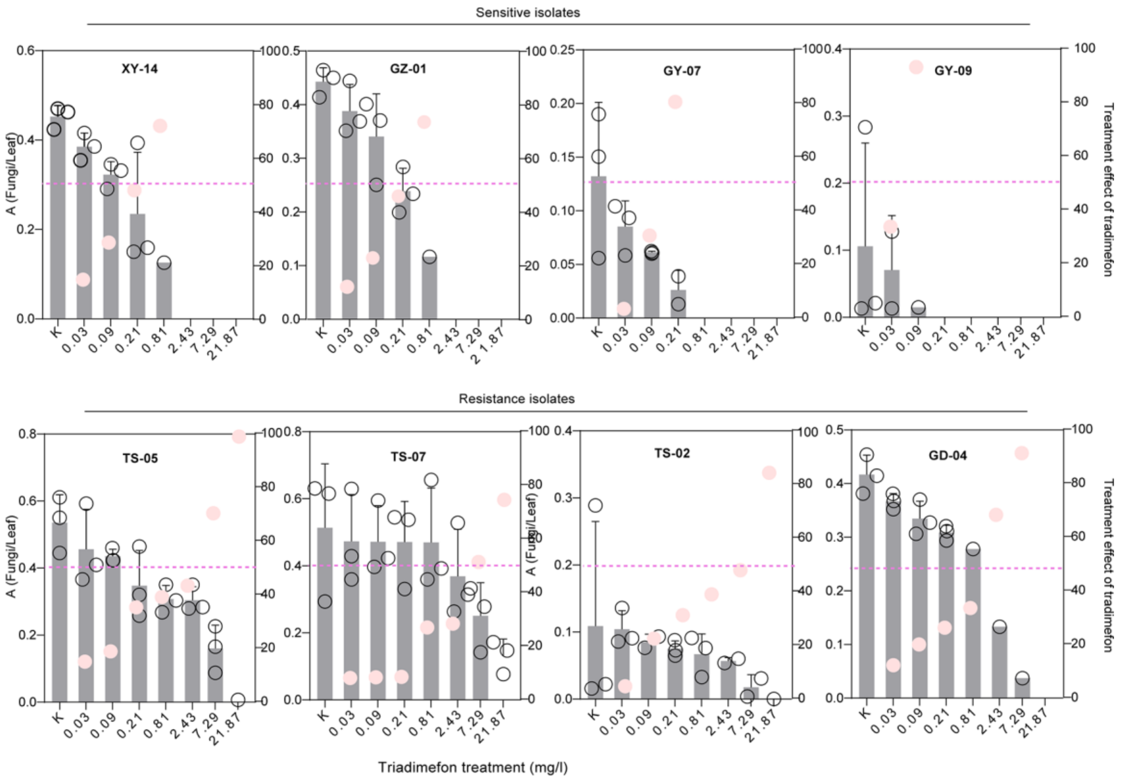

Supplementary Fig. 33. EC_{50} measurement of isolates against triadimefon.

$A_{(Fungi/Leaf)}$: the proportion of sporulating area of the leaf segments; Relative inhibition rate: $[A_{(Fungi/Leaf)0}-A_{(Fungi/Leaf)}]/A_{(Fungi/Leaf)0}$. $A_{(Fungi/Leaf)0}$: the proportion of sporulating area of the leaf segments without the treatment of triadimefon. The absence of dose data indicates that there is no spore growth under the treatment of this concentration of triadimefon. The ratio of sporulating area of the leaf segments was zero. Data are means ± SD (n = 3). Source data are provided as a Source Data file.

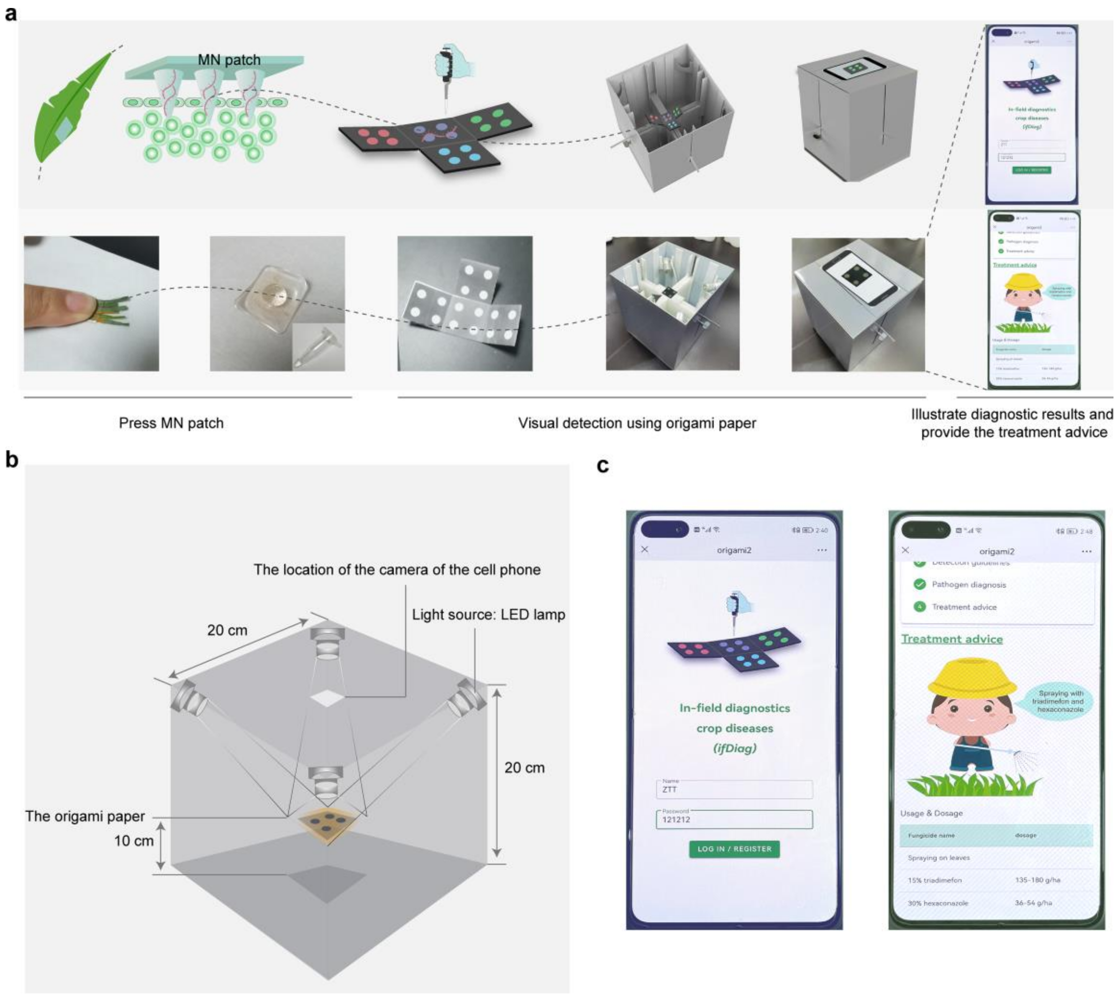

Supplementary Fig. 34. Integrated assay for in-field detection of wheat diseases.

Scheme of the process flows of the in-field test for crop diseases **(**a**)**. The apparatus (b) and app (c) for assisting the photographing of origami papers. The portable apparatus provides the light source and the origami folding platform. The origami paper is placed on the folding platform at the center of the apparatus, and the camera lens of the cell phone is positioned 10-cm above the origami paper for capturing photos. Four LED lights ensure a consistent light source.

.

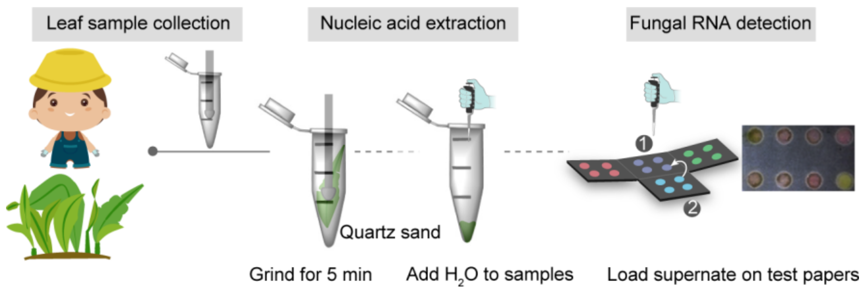

Supplementary Fig. 35. Rapid nucleic acid extraction using quartz sand.

Nucleic acid extraction from wheat leaves was proceeded using quartz sand, and the process was finished within 5 min.

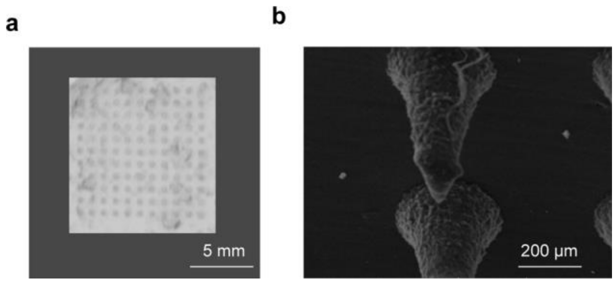

Supplementary Fig. 36. Photograph and scanning electron microscopy image of a MN patch. A photograph (**a**) and a scanning electron microscopy image (**b**) of the MN patch. The MN patch contains a 11 × 11 microneedle array. The height and base of needle are 600 μm and 300 μm. The spacing tip to tip is 600 μm.

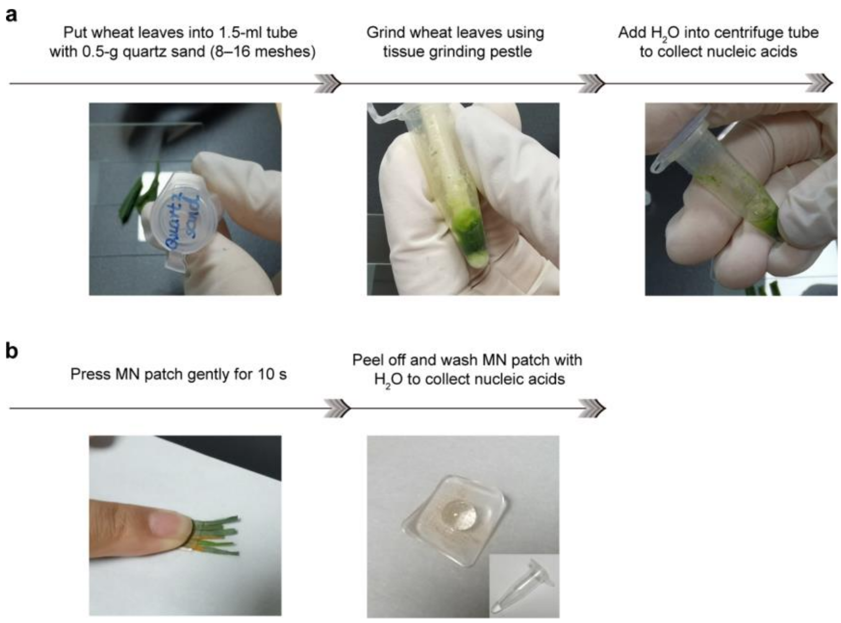

Supplementary Fig. 37. Nucleic acid extraction using quartz sand and MN patch.

Steps of quartz sand-based (**a**) and microneedle (MN) patch-based (**b**) nucleic acid extraction approaches from infected wheat leaves. **a**, Wheat leaves were grinded in 1.5 mL centrifuge tube with quartz sand (8–16 meshes), and collected in H_2O. **b,** MN patch was gently pressed on wheat leaves, peeled off to collect nucleic acids.

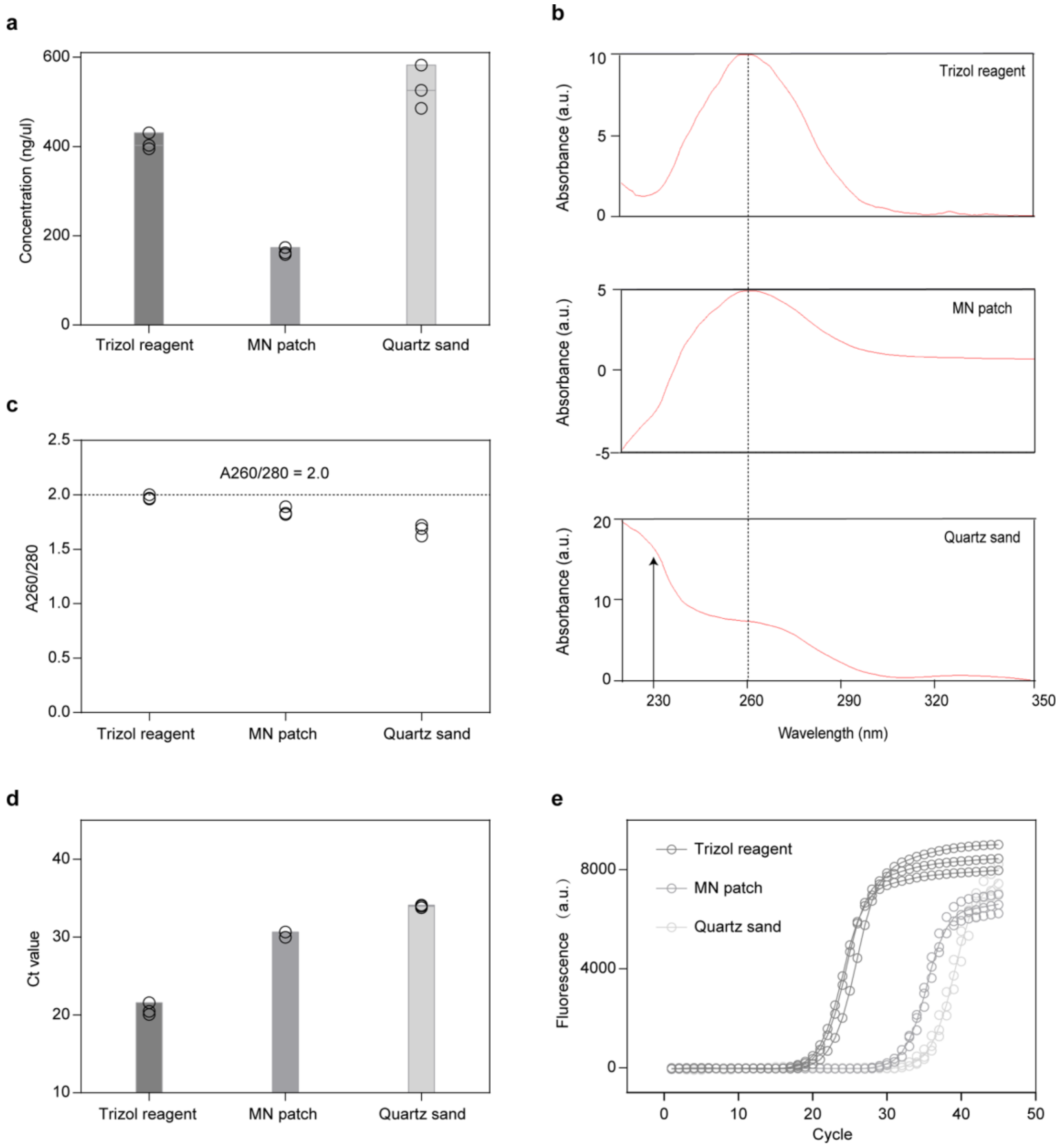

Supplementary Fig. 38. Comparison of extraction efficiency and purity using Trizol, MN patch, and quartz sand.

Concentration (**a**), absorbance spectra (**b**), A260/A280 (**c**) of extractive from *Pst* infected wheat leaves using Trizol reagent, MN patch, and quartz sand. *Ct* values (**d**) and amplification curves (**e**) for detecting *Pst* ITS RNAs in extractive from *Pst* infected wheat leaves using Trizol reagent, MN patch, and quartz sand. Data in **a, c** and **d** are means (n = 3). Extraction using quartz sand yielded the highest amount of extractive, and MN patch yielded about half of the amount of extractive compared to Trizol-based method (Supplementary Fig. 29a). However, the absorbance spectra indicated that quartz sand-based extraction are heavily polluted with polysaccharides (Supplementary Fig. 29b). The use of MN yielded a A260/A280 close to that use of Trizol (Supplementary Fig. 29c), indicating that MN patch extraction allow to extract nucleic acid with sight impurity such as proteins and polysaccharides. All of the samples extracted using MN patch, Trizol and quartz sand can be

positively detected with *Pst* RNAs (Supplementary Fig. 29d). High *Ct* value testing quartz sand-based extracted sample may be due to the abundance of contaminants. The MN method extracted less nucleic acid than the Trizol-based method. This may be due to the difference of sampling volumes. Sampling volumes for MN patch is about 1% of that for the Trizol extraction method. Source data are provided as a Source Data file.

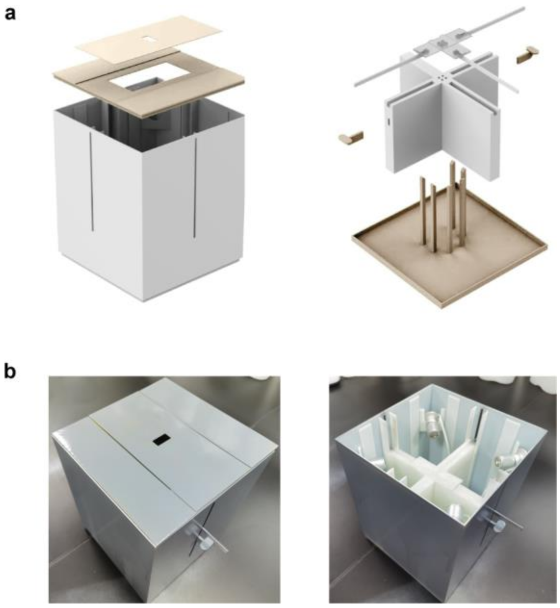

Supplementary Fig. 39. Smartphone attachment designed for colorimetric detection of fungal pathogens.

The model (**a**) and 3D-printed (**b**) portable device used for the colorimetric detection of fungal RNAs. The attachment was designed using rhino6 and made using Lite 800 (Union Tech., Shanghai, China). The device consisted of three components, four light-emitting diodes (LEDs) (i), a solution tank (ii), and a paper-folding assistant unit (iii). (i) LEDs were used to provide a stable light source for paper visualization. (ii) Saturated $NaNO_3$ solution was placed in the tank to maintain a suitable humid condition to avoid sample evaporation. (iii) a paper-folding assistant unit allowed to finish the paper-folding processes by simple pull operations, and rendering the test finished in the chambers. The device is 30 cm long, 18 cm wide and 22 cm deep, and weighs ~0.3 kg.

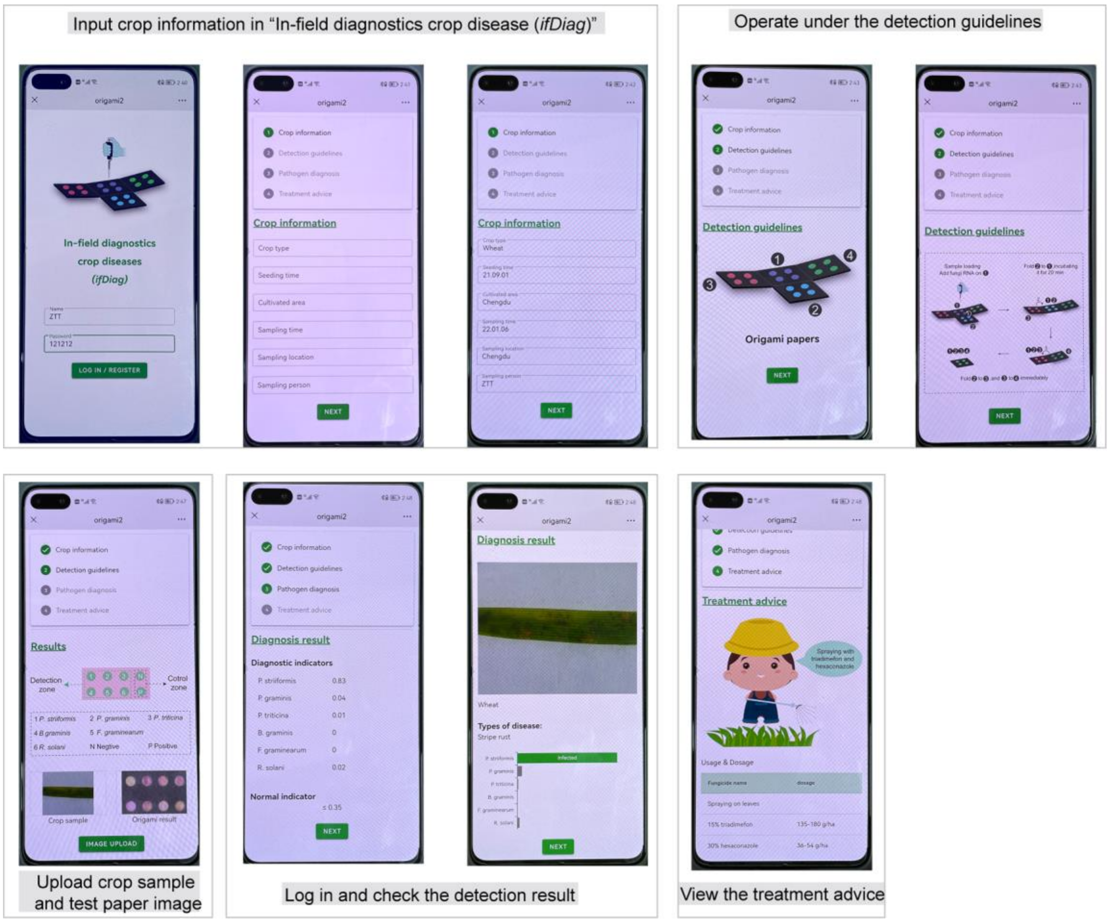

Supplementary Fig. 40. A smartphone app for in-field diagnostics of crop diseases.

The app, named "In field diagnostics crop disease (*ifDiag*)", consists of crop information record (i), detection guidelines (ii), pathogen diagnosis (iii) and treatment advice (iv). (i) Sign in and input crop information including crop type, seeding time, cultivated areas, and sampling information in *ifDiag*. (ii) Detect fungi infected wheat leaves under the detection guidelines. (iii) Upload crop diagnosis results (iii) and offer treatment advice based on the detection results in *ifDiag*.

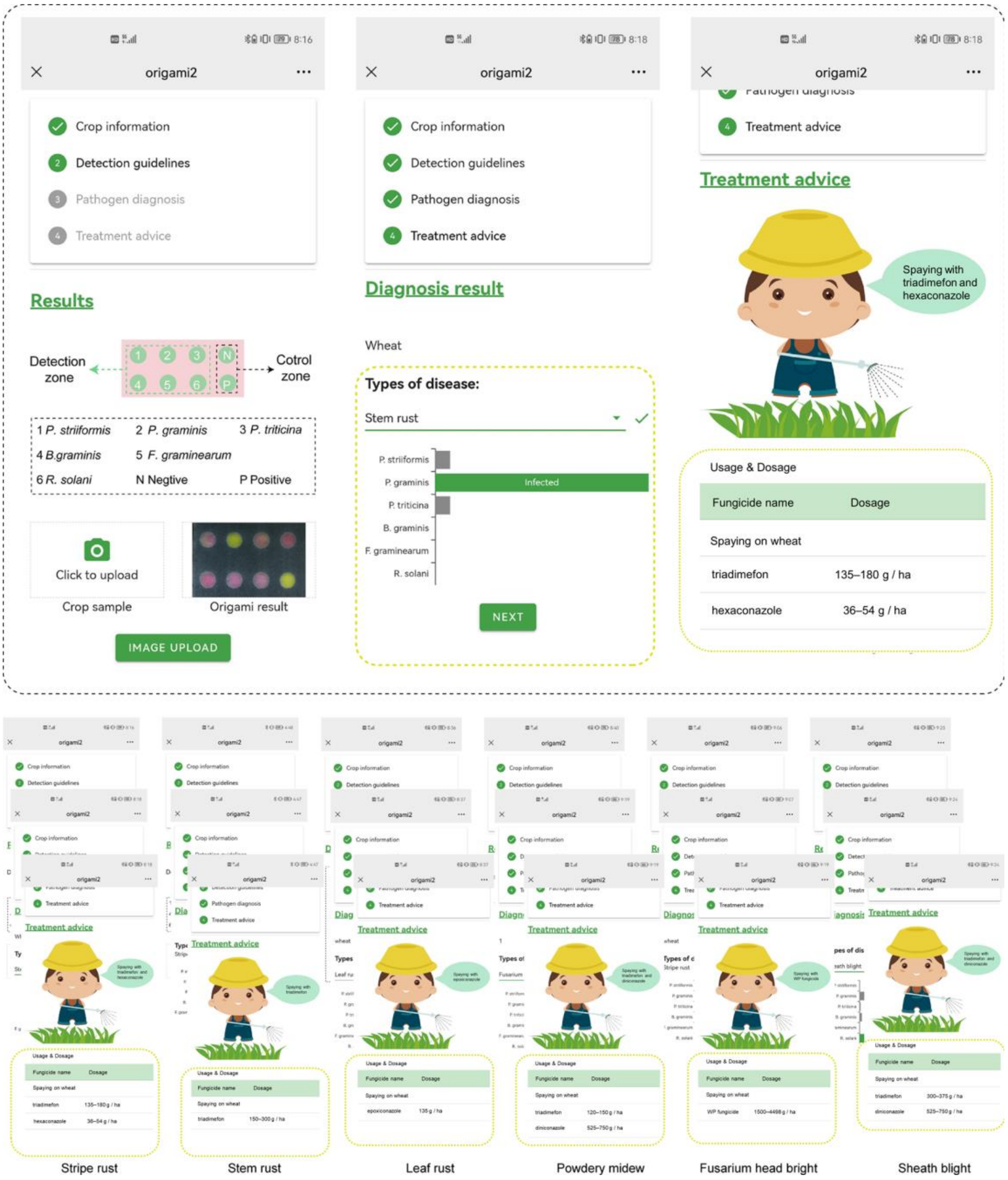

Supplementary Fig. 41. Demonstration of intelligent diagnosis enabled by *ifDiag* app. Origami papers were used to test wheat samples separately infected with *Pst, Pgt, Pt, Bgt, Fg* and *Rc*, and imaged using smartphone. The instruction for treating crop infection by *Pst, Pgt, Pt, Bgt, Fg* and *Rc* has been programmed in the app. Besides offering the guideline for operation, *ifDiag* app provides the diagnostic result, and based on the infected pathogens, allows to tell the user how to treat the infection, such as which fungicide to choose and how many dosages to be used, based on the diagnostic result. Based on the tool, the end-user without any knowledge about phytopathology can be instructed to precisely treat the crop infection.

Supplementary Note 1. Optimization of origami papers

Paper-based tests including microfluidic paper-based analytical devices (μPADs) and origami papers facilitated on-site detection via loading detection reagents on papers and integrating each detection reaction on a paper[7,8]. Paper-based tests are cheap and easy-to-prepare. The performance of paper-based tests would be influenced by the color nonuniformity caused by reagent diffusion in the paper[9-13]. In the test using the assay, the colorimetric change in the circular detection zones is not so uniform, it may be resulted from the diffusion kinetics and non-uniformity related to the movement of the reagent or sample from the page to the contacted page activated by folding. Physical contact via paper folding cannot uniformize the contact between two pages due to the uneven pressure. We expect that rapid diffusion of samples or reagents can alleviate the effect of non-uniform contact on constant colour change via efficiently spread from tightly contacted locality with higher concentration of samples or reagents (such as the central position) to less contacted locality. For paper-based detection, diffusion is highly affected by the porosity and hydrophilicity of the paper. We chose urea cleavage catalyzed by urease as the reaction to investigate the effect of the porosity and hydrophilicity of the paper on sensitivity and the consistency as the reaction can be recorded by colour change (Supplementary Fig. 11). We loaded urea and phenol red at one paper, and urease at another page, and used the folding strategy to initiate the cleavage of urea and colour presence. We used GPR values to quantify the colour change of the paper, and the ratio of GPRs in the absence of the presence of urease (B/S ratio) to indicate the response and sensitivity on the paper. We used the standard deviation of GPR values in a replicated 5 test with the presence of urease to indicate the consistency of the test. As the cleavage of urea catalyzed via urease increases pH and turns phenol red from red to yellow, thus mainly inducing the gray value change in the green channel. The standard deviation of the gray value in the green channel in each pixel in the circular detection zones was used to estimate the uniformity of the colorimetric response.

Before examining the effect of porosity and hydrophilicity of the paper, we tested signal response in the circular detection zones with different sizes. In the test, 5 μL sample was loaded on the origami papers. Small size of the circular detection zones shortens the diffusion distance, but with a droplet of 5 μL sample, we found that the detection site of 1.5 mm-diameter cannot load the samples. Compared to that with a diameter of 3.5 mm, the circular detection zone with increased diameter to

be 5.5 to 7.5 mm yielded a dramatically reduced B/S ratio (Supplementary Fig. 11c). This may because that the circular detection zones with large area dilute the reagents including urease, urea and phenol red. Whatman No. 1 filter papers was used as the origami papers in the assay. We further tested the filter papers with pore size ranged from 12.5 μm to 22.5 μm. Using the filter papers with larger pore size increased the B/S ratio towards urease, and yielded a higher uniformity and consistency of colorimetric response in a replicated 5 test (Supplementary Fig. 11e, f and g). The result showed that filter papers with large pore size facilitate the reagent diffusion and would benefit both the sensitivity and the robustness of paper-based colorimetric detection. Surfactants have been used to increase the hydrophilicity and facilitate reagent diffusion[14]. We investigated the effects of three typical surfactant, cationic surfactant cetrimonium bromide (CTAB), anionic surfactant sodium dodecyl sulfate (SDS), and non-ionic surfactant Tween 20 on detecting urease-based colorimetric detection. 1 μM surfactant was dropped onto the circular detection zones and dried at room temperature before testing. The addition of CTAB and Tween 20 dramatically improved the B/S ratio (Supplementary Fig. 11i), and the consistency and uniformity of colorimetric response in a replicated 5 test have also improved compared that without pre-loading of surfactant (Supplementary Fig. 11 j and k). We further investigated the performance of the assay for diagnosing *Pst* RNA using the optimized origami modified with CTAB and with a pore size of 22.5 μm (Supplementary Fig. 12a and b). The result shows that, compare to the usage of original papers, the standard deviation of GPR has dramatically been reduced (from 14.42 to 5.77 (the CTAB-treated origami paper), and 5.64 (the origami paper with a pore size of 22.5 μm)) (Supplementary Fig. 12c), indicating that the optimization of pore size and the addition of surfactant yields an improved color homogeneity for diagnosing *Pst* RNA. These results demonstrated that increasing the diffusion kinetic can facilitate the paper-based colorimetric detection with improved sensitivity, consistency and color uniformity.

Supplementary Note 2. Defense response of wheat triggered by the dead *Pst* spores

The defense response of wheat triggered by the dead *Pst* spores was investigated. The inoculated leaves with dead or viable *Pst* spores were harvested at 12, 24 and 48 h post inoculation. The expression of marker genes involved in plant defense signaling pathways in wheat, including PR1 (Pathogenesis-related protein 1) and PR2 (a beta-1,3-endoglucanase) were quantified by RT-qPCR.

The expression of PR1 and PR2 was slightly improved via the inoculation with the inactivate dead spores, and was much lower than that inoculated with viable spores (**Supplementary Fig. 29**).

We then tested whether the dead *Pst* spores affect the infection. After two days of dead *Pst* spore inoculation, the viable *Pst* spores were sequentially inoculated. The inoculated leaves were harvested at 5-, 7-, 11-, and 13-day post inoculation after the second inoculation. Then, biomass of *Pst* between pre-treatment with the dead *Pst* spores and control group was quantified. The pre-inoculation of the dead spores did not significantly change *Pst* biomass after 12 h-, 24 h-, 48 h-infection with the viable spores (Supplementary Fig. 30).

These results collectively indicated that challenged non-viable pathogens did not elicit a defence response that efficiently inhibit viable pathogen growth. Plants have evolved a two-layered innate immune system, which are defined as pathogen-associated molecular pattern (PAMP)-triggered immunity (PTI) and effector-triggered immunity (ETI)[15]. However, for the non-viable spores, the intense ETI was hardly to be excited. Cell wall of *Pst* spores includes polysaccharide, protein, chitin and other substances. Chitin is sensed by the immune system as a PAMP through specific membrane-bound receptors or named pattern recognition receptors induce weak immune response[16]. But the contact of chitin in the cell wall of spores is expected to be hindered by the cuticle of the leaf. Without germination, dead spores may exert weak PTI that can hardly support a defence response affecting sequential viable pathogen infection.

Supplementary Note 3. Differentiation of single-nucleotide mutations using DProbes

Differentiation of single-nucleotide mutations in the input RNA using TMSD reaction is based on competitive hybridization[17,18]. Alternative to the hybridization recognition designed in traditional DNA assays and PCR, competitive hybridization alleviates the mismatched hybrids via designing a blocker sequence (herein, the *Trans* strand serves as the blocker), not via elevating hybridization temperature[1,2]. The DProbe contains a couple of forward and reverse toehold domains at the terminal site. The forward toehold is the domain in the *Cis* strand that hybridizes with the input RNA while not the *Trans* strand. Reversely, the reverse toehold is the domain that in the *Cis* strand that

hybridizes with the *Trans* strand while not the input RNA. Therefore, competitive binding of the input RNA with the *Cis* strand in the DProbe, is facilitated by the forward toehold, while hindered via disrupting the reverse toehold. The net effect of the strand displacement process is determined by the formation of new base pairs within the forward toehold and the disruption of the former base pairs within the reverse toehold[19]. To maximize the discrimination between the target input RNA and the non-target input RNA with single-nucleotide difference, the TMSD reaction should be optimized via turning the toehold length of the DProbe, to main hybridization stability of the DProbe, that allows to block non-specific hybridization induced by the non-target input RNA while still form the hybridization of target input RNA-the *Cis* strand based on the different affinity between non-target input RNA and target input RNA towards the *Cis* strand.

Supplementary Table 1. Oligonucleotides used for detecting *Pst*.

Name	Sequence (5'-3')	Annotation
Cis strand-22-1	GATAGGCTCGATGATTCACTGAATTCTGC	Forward toehold and reverse toehold are marked in pink and green, respectively. The mismatch bases are framed in black.
Cis strand-22-2	GATAGGCTCCATTGAAAATAATTAATCAA	
Cis strand-22-3	GATAGGCCGTCTACTCAATTGCAATGATT	
Cis strand-22-4	GATAGGCTACAGCAGCACTCAACATCCAT	
Cis strand-35-10	GATAGGCAATAAAGAAGTTACGTCTACTCAATTGCAATGATT	
Cis strand-35-15	GATAGGCAATAAAGAAGTTACGTCTACTCAATTGCAATGATT	
Cis strand-35-20	GATAGGCAATAAAGAAGTTACGTCTACTCAATTGCAATGATT	
Trans strand-22-1	CAGTGAATCATCGACCCTATC	
Trans strand-22-2	TTATTTTCAATGGACCCTATC	
Trans strand-22-3	CAATTGAGTAGACGCCCTATC	
Trans strand-22-4	TGAGTGCTGCTGTACCCTATC	
Trans strand-35-10	ATTGAGTAGACGTAACTTCTTTATTCCCTATC	
Trans strand-35-15	GTAGACGTAACTTCTTTATTCCCTATC	
Trans strand-35-20	CGTAACTTCTTTATTCCCTATC	
Cis-labeled	6FAM'-GATAGGCCCCTTTGGTATTCCAAAGGGCATGCCTGTTCGAGC	Forward toehold and reverse toehold are marked in pink and green, respectively. The mismatch bases are framed in black.
Trans-labeled	CCCTTTGGAATACCAAAGGGCCCTATC-BHQ1	
Cis-F134	6FAM'-GATAGGCTTGCGTTCGGGACATCAAAAACTACATCCGTACCG	
Trans-5 nt	CGGATGTAGTTTTTGATGTCCCGAACGCAACCCTATC-BHQ1	
Trans-9 nt	TGTAGTTTTTGATGTCCCGAACGCAACCCTATC-BHQ1	
Trans-13 nt	GTTTTTGATGTCCCGAACGCAACCCTATC-BHQ1	
Trans-17 nt	TTGATGTCCCGAACGCAACCCTATC-BHQ1	
Trans-21 nt	TGTCCCGAACGCAACCCTATC-BHQ1	
Trans-25 nt	CCGAACGCAACCCTATC-BHQ1	

Supplementary Table 2. Oligonucleotides used for electrophoretic analysis (Fig. 1c).

Name	Sequence (5'-3')	Annotation
Target RNA	GCTCGAACAGGCATGCCCTTTGGAATACCAAAGGG	Forward toehold and reverse toehold were marked in pink and green, respectively. The mismatch bases were framed in black.
Cis strand	GATAGGCCCCTTTGGTATTCCAAAGGGCATGCCTGTTCGAGC	
Trans strand	TTTTTTTTTTTTTTTTTTTTTTTTTTTTTTCCCTTTGGAATACCAAAGGGCCCTATC	
Cis-F134	GATAGGCTTGCGTTCGGGACATCAAAAACTACATCCGTACCG	Forward toehold and reverse toehold were marked in pink and green, respectively. The mismatch bases were framed in black.
Trans-5 nt	TTTTTTTTTTTTTTTTTTTTTTTTTTTTTTCGGATGTAGTTTTTGATGTCCCGAACGCAACCCTATC	
Trans-9 nt	TTTTTTTTTTTTTTTTTTTTTTTTTTTTTTTGTAGTTTTTGATGTCCCGAACGCAACCCTATC	
Trans-13 nt	TTTTTTTTTTTTTTTTTTTTTTTTTTTTTTGTTTTTGATGTCCCGAACGCAACCCTATC	
Trans-17 nt	TTTTTTTTTTTTTTTTTTTTTTTTTTTTTTTTGATGTCCCGAACGCAACCCTATC	
Trans-21 nt	TTTTTTTTTTTTTTTTTTTTTTTTTTTTTTTGTCCCGAACGCAACCCTATC	
Trans-25 nt	TTTTTTTTTTTTTTTTTTTTTTTTTTTTTTCCGAACGCAACCCTATC	

Supplementary Table 3. Oligonucleotides used for detecting *Pst, Pgt, Pt, Bgt, Fg Rc, P. syringae* *pv. tomato* DC3000, and BSMV.

Name	Pathogenic	Sequence (5'-3')
Cis strand	*Pst*	GATAGGCAATAAAGAAGTTACGTCTACTCAATTGCAATGATT
	Pgt	GATAGGCATTAAGAATGTTACGTATACTCAATTGCAATGACC
	Pt	GATAGGCATAAAGAATGCCACGTATACTTAATCACAATGATT
	Bgt	GATAGGCGCGAAATGCGATAAGTAATGTGAATTGCAGAATTT
	Fg	GATAGGCCGATAAGTAATGTGAATTGCAGAATTCAGTGAATC
	Rc	GATAGGCAATACATAAAATCTTATATATTTAATCAGAATGTA
	DC3000-1	GATAGGCTACACCTATGTATTCAGTGTAAGATAACCATCTTA
	DC3000-2	GATAGGCGCTATCACCCACTATGGCCGCCCTTTCCAGAGCGT
	BSMV-1	GATAGGCACCTACTCACGATTGGCAGTTGATCACAAGCCTC
	BSMV-2	GATAGGCGTTTCCAATTCAGGCATCGTTTTCAAGTTCGATT
Trans strand	*Pst*	GTAGACGTAACTTCTTTATTCCCTATC
	Pgt	GTATACGTAACATTCTTAATCCCTATC
	Pt	GTATACGTGGCATTCTTTATCCCTATC
	Bgt	CATTACTTATCGCATTTCGCCCCTATC
	Fg	GCAATTCACATTACTTATCGCCCTATC
	Rc	ATATATAAGATTTTATGTATTCCCTATC
	DC3000-1	TACACTGAATACATAGGTGTACCCTATC
	DC3000-2	GCGGCCATAGTGGGTGATAGCCCCTATC
	BSMV-1	AACTGCCAATCGTGAGTAGGTCCCTATC
	BSMV-2	AAACGATGCCTGAATTGGAAACCCCTATC

Supplementary Table 4. Comparison of results for detecting *Pst* infection in samples using the colorimetric assay and qPCR.

qPCR	Number	Colorimetric assay	Number	Agreement (%)
Negative	21	Negative	20	95.3
		Positive	1	
Positive	11	Negative	1	91.0
		Positive	10	

Supplementary Table 5. qPCR and RT-qPCR primers for detecting *Pst,* pathogenesis-related genes and fungal biomass.

Primers	Sequence (5'-3')	Description
PR1-F	GAGAATGCAGACGCCCAAGC	RT-qPCR primer for the estimation of the expression level of pathogenesis-related genes
PR1-R	CTGGAGCTTGCAGTCGTTGATC	
PR2-F	AGGATGTTGCTTCCATGTTTGCCG	
PR2-R	AAGTAGATGCGCATGCCGTTGATG	
PstEF1-F	TTCGCCGTCCGTGATATGAGACAA	qPCR primer for *Pst* internal control gene PstEF1-α
PstEF1-R	ATGCGTATCATGGTGGTGGAGTGA	
TaEF-F	TGGTGTCATCAAGCCTGGTATGGT	qPCR primer for wheat internal control gene TaEF1-α
TaEF-R	ACTCATGGTGCATCTCAACGGACT	
ITS-F	TTCGCCGTCCGTGATATGAGACAA	qPCR primer for quantification of *Pst* by targeting the ITS sequence
ITS-R	ATGCGTATCATGGTGGTGGAGTGA	

Supplementary Table 6. EC_{50} and EC_{90} measurement of eight isolates against triadimefon.

Isolate	Province	EC_{50} (mg/L)	EC_{90} (mg/L)	Toxicity regression equation*	R^2	Resistance Index
XY-14	Shaanxi	0.14	0.41	Y=7.36+2.78X	0.86	0.74
GZ-01	Guizhou	0.15	0.42	Y=7.36+2.87X	0.87	0.79
TS-05	Gansu	1.82	11.45	Y=5.09+0.96X	0.89	9.55
TS-07	Gansu	6.65	364.56	Y=4.39+0.74X	0.95	34.99
GY-07	Yunnan	0.10	0.19	Y=9.58+4.60X	0.95	0.53
GY-09	Yunnan	0.04	0.07	Y=12.96+5.69X	0.98	0.21
TS-02	Gansu	3.13	135.80	Y=4.61+0.783X	0.92	16.50
GD-04	Qinghai	1.43	2.03	Y=5.70+1.89X	0.89	7.50

* X: Log_{10}([triadimefon]); Y: Relative inhibition rate.

Supplementary Table 7. Six predominant wheat diseases caused by fungal pathogens and treatment advices.*

**Fungal pathogen**	**Crop disease**	**Disease management**
Pst	Stripe rust	1. Spraying on leaves with 15% triadimefon, the dosage is 135–180 g (effective ingredient) / ha.<br>2. Spraying on leaves with 30% hexaconazole, the dosage is 36–54 g (effective ingredient) / ha.
Pgt	Stem rust	Spraying on the wheat with triadimefon, the dosage is 150–300 g (effective ingredient) / ha.
Pt	Leaf rust	Spraying on the wheat with epoxiconazole, the dosage is 135 g (effective ingredient) / ha.
Bgt	Powdery mildew	1. Spraying on wheat with triadimefon, the dosage is 120–150 g (effective ingredient) / ha.<br>2. Spraying on the wheat with diniconazole (12.5%), the dosage is 525–750 g (effective ingredient) / ha.
Fg	Fusarium head blight	Spraying on the wheat with WP fungicide (carbendazim 24% + triadimefon 9%), the dosage is 1500–4498 g / ha.
Rc	Sheath blight	1. Spraying on the wheat with diniconazole (12.5%), the dosage is 525–750 g (effective ingredient) / ha.<br>2. Spraying on the wheat with triadimefon (25%), the dosage is 300–375 g (effective ingredient) / ha.

*The instruction for treating wheat infection by *Pst, Pgt, Pt, Bgt, Fg* and *Rc* was based on technical specification accommodated by General Administration of Quality Supervision, Inspection and Quarantine of the People's Republic of China.

Supplementary references

1. Li Q., Luan G., Guo Q., Liang J. A new class of homogeneous nucleic acid probes based on specific displacement hybridization. *Nucleic Acids Res.* **30**, e5-e5 (2002).
2. Altan-Bonnet G., Kramer F. R. Robust sequence discrimination. *Nat. Chem.* **4**, 155-157 (2012).
3. Ono A*., et al.* Specific interactions between silver(i) ions and cytosine–cytosine pairs in DNA duplexes. *Chem. Commun.*, 4825-4827 (2008).
4. Mazzei L., Cianci M., Gonzalez Vara A., Ciurli S. The structure of urease inactivated by Ag(i): a new paradigm for enzyme inhibition by heavy metals. *Dalton Transactions* **47**, 8240-8247 (2018).
5. Carrilho E., Martinez A. W., Whitesides G. M. Understanding wax printing: A simple micropatterning process for paper-based microfluidics. *Anal. Chem.* **81**, 7091-7095 (2009).
6. Tooei M. H. D. H., Mianroodi J. R., Norouzi N., Khajooeizadeh A. An innovative implementation of Circular Hough Transform using eigenvalues of Covariance Matrix for detecting circles. In: *Proceedings ELMAR-2011* (2011).
7. Noviana E*., et al.* Microfluidic paper-based analytical devices: From design to applications. *Chem. Rev.* **121**, 11835-11885 (2021).
8. Liu H., Crooks R. M. Three-dimensional paper microfluidic devices assembled using the principles of origami. *J. Am. Chem. Soc.* **133**, 17564-17566 (2011).
9. Evans E., Gabriel E. F. M., Coltro W. K. T., Garcia C. D. Rational selection of substrates to improve color intensity and uniformity on microfluidic paper-based analytical devices. *Analyst* **139**, 2127-2132 (2014).
10. Verma M. S*., et al.* Sliding-strip microfluidic device enables ELISA on paper. *Biosens. Bioelectron.* **99**, 77-84 (2018).
11. Dong T., Wang G. A., Li M. W., Li F. Systematic investigation of a quantitative paper-based DNA reader (qPDR) for distance-based quantification of nucleic acids and mercury ions. *Anal. Methods* **11**, 5376-5380 (2019).
12. Chung S*., et al.* Norovirus detection in water samples at the level of single virus copies per microliter using a smartphone-based fluorescence microscope. *Nat. Protoc.* **16**, 1452-1475 (2021).
13. Soda Y., Robinson K. J., Cherubini T. J., Bakker E. Colorimetric absorbance mapping and

quantitation on paper-based analytical devices. *Lab Chip* **20**, 1441-1448 (2020).

14. Xue Y.-Y.*, et al.* Development of a paper-based microfluidic analytical device by a more facile hydrophobic substrate generation strategy. *Anal. Biochem.* **525**, 100-106 (2017).
15. Jones J. D. G., Dangl J. L. The plant immune system. *Nature* **444**, 323-329 (2006).
16. Klauser D., Flury P., Boller T., Bartels S. Several MAMPs, including chitin fragments, enhance AtPep-triggered oxidative burst independently of wounding. *Plant Signal Behav.* **8**, e25346 (2013).
17. Zhang D. Y., Chen S. X., Yin P. Optimizing the specificity of nucleic acid hybridization. *Nat. Chem.* **4**, 208-214 (2012).
18. Wu L. R.*, et al.* Continuously tunable nucleic acid hybridization probes. *Nat. Methods* **12**, 1191-1196 (2015).
19. Hong F.*, et al.* Precise and programmable detection of mutations using ultraspecific riboregulators. *Cell* **180**, 1018-1032.e1016 (2020).
