# Supplementary material for: Precise in-field molecular diagnostics of crop diseases by smartphone-based mutation-resolved pathogenic RNA analysis
Source: Nat Commun. 2023 Jul 19;14:4327. doi: 10.1038/s41467-023-39952-x (PMC10356797; doi:10.1038/s41467-023-39952-x)
Supplement: Supplementary file 2 — Reporting Summary [file 41467_2023_39952_MOESM2_ESM.pdf]

nature portfolio

Corresponding author(s): Ruijie Deng

Last updated by author(s): Jun 15, 2023

Reporting Summary

Nature Portfolio wishes to improve the reproducibility of the work that we publish. This form provides structure for consistency and transparency in reporting. For further information on Nature Portfolio policies, see our Editorial Policies and the Editorial Policy Checklist.

Statistics

For all statistical analyses, confirm that the following items are present in the figure legend, table legend, main text, or Methods section.

n/a	Confirmed	
☐	☒	The exact sample size (*n*) for each experimental group/condition, given as a discrete number and unit of measurement
☐	☒	A statement on whether measurements were taken from distinct samples or whether the same sample was measured repeatedly
☐	☒	The statistical test(s) used AND whether they are one- or two-sided<br>*Only common tests should be described solely by name; describe more complex techniques in the Methods section.*
☒	☐	A description of all covariates tested
☒	☐	A description of any assumptions or corrections, such as tests of normality and adjustment for multiple comparisons
☐	☒	A full description of the statistical parameters including central tendency (e.g. means) or other basic estimates (e.g. regression coefficient) AND variation (e.g. standard deviation) or associated estimates of uncertainty (e.g. confidence intervals)
☐	☒	For null hypothesis testing, the test statistic (e.g. *F*, *t*, *r*) with confidence intervals, effect sizes, degrees of freedom and *P* value noted<br>*Give P values as exact values whenever suitable.*
☒	☐	For Bayesian analysis, information on the choice of priors and Markov chain Monte Carlo settings
☒	☐	For hierarchical and complex designs, identification of the appropriate level for tests and full reporting of outcomes
☒	☐	Estimates of effect sizes (e.g. Cohen's *d*, Pearson's *r*), indicating how they were calculated

Our web collection on statistics for biologists contains articles on many of the points above.

Software and code

Policy information about availability of computer code

Data collection	All absorbance data were collected using Gen5 CHS 3.08. Cycle threshold values for RNA and DNA level determination were determined using CFX96 Touch Real-Time PCR Detection System.
Data analysis	Figures were created using Origin 2019. Welch's t-test was used for all statistical comparisons, and calculated by SPSS 25. ROC curves were produced using GraphPad Prism 8.

For manuscripts utilizing custom algorithms or software that are central to the research but not yet described in published literature, software must be made available to editors and reviewers. We strongly encourage code deposition in a community repository (e.g. GitHub). See the Nature Portfolio guidelines for submitting code & software for further information.

Data

Policy information about availability of data

All manuscripts must include a data availability statement. This statement should provide the following information, where applicable:

- Accession codes, unique identifiers, or web links for publicly available datasets
- A description of any restrictions on data availability
- For clinical datasets or third party data, please ensure that the statement adheres to our policy

All data generated or analyzed during this study are included in this article and its Supplementary Information/Source Data file. Source data are provided with this paper.

Human research participants

Policy information about studies involving human research participants and Sex and Gender in Research.

Reporting on sex and gender	The study did not involve human research participants, thus not provide sex and gender information.
Population characteristics	The study did not involve human research participants, thus not provide information about population characteristics.
Recruitment	The study did not involve human research participants.
Ethics oversight	The study did not involve human research participants.

Note that full information on the approval of the study protocol must also be provided in the manuscript.

Field-specific reporting

Please select the one below that is the best fit for your research. If you are not sure, read the appropriate sections before making your selection.

[x] Life sciences [ ] Behavioural & social sciences [ ] Ecological, evolutionary & environmental sciences

For a reference copy of the document with all sections, see nature.com/documents/nr-reporting-summary-flat.pdf

Life sciences study design

All studies must disclose on these points even when the disclosure is negative.

Sample size	No statistical method was used to predetermine sample sizes. Our sample sizes are similar to those generally used in the field.
Data exclusions	No data were excluded.
Replication	For the majority of the studies three or more repeats were done, to ensure reproducibility. The replication numbers are listed in legends and In Results.
Randomization	Wheat leaf samples for estimating the accuracy of the colorimetric assay were randomly picked to test (Fig. 2d and 2e). Other samples were not randomized.
Blinding	Blinding of wheat samples were performed before accuracy test using the colorimetric assay and qPCR (Fig. 2d and 2e).

Reporting for specific materials, systems and methods

We require information from authors about some types of materials, experimental systems and methods used in many studies. Here, indicate whether each material, system or method listed is relevant to your study. If you are not sure if a list item applies to your research, read the appropriate section before selecting a response.

Materials & experimental systems

n/a	Involved in the study	
x		Antibodies
x		Eukaryotic cell lines
x		Palaeontology and archaeology
	x	Animals and other organisms
x		Clinical data
x		Dual use research of concern

Methods

n/a	Involved in the study	
x		ChIP-seq
x		Flow cytometry
x		MRI-based neuroimaging

Animals and other research organisms

Policy information about studies involving animals; ARRIVE guidelines recommended for reporting animal research, and Sex and Gender in Research

Laboratory animals	The study did not involve laboratory animals.
Wild animals	The study did not involve laboratory animals.

Reporting on sex	The study did not involve laboratory animals.
Field-collected samples	During February to May 2021, wheat leaf samples were collected from fields from five provinces in China, including Gansu, Guizhou, Qinghai, Shaanxi and Yunnan.
Ethics oversight	No ethical approval or guidance was required as no animal involved in the study.

Note that full information on the approval of the study protocol must also be provided in the manuscript.
